# Small RNA sequencing reveals a role for sugarcane miRNAs and their targets in response to *Sporisorium scitamineum* infection

**DOI:** 10.1186/s12864-017-3716-4

**Published:** 2017-04-24

**Authors:** Yachun Su, Yuye Zhang, Ning Huang, Feng Liu, Weihua Su, Liping Xu, Waqar Ahmad, Qibin Wu, Jinlong Guo, Youxiong Que

**Affiliations:** Key Laboratory of Sugarcane Biology and Genetic Breeding, Ministry of Agriculture, Fujian Agriculture and Forestry University, Fuzhou, 350002 China

**Keywords:** *Saccharum* spp., *Sporisorium scitamineum*, High-throughput sequencing, miRNA, Function prediction of target genes, qRT-PCR

## Abstract

**Background:**

Sugarcane smut caused by *Sporisorium scitamineum* leads to a significant reduction in cane yield and sucrose content. MicroRNAs (miRNAs) play an important role in regulating plant responses to biotic stress. The present study was the first to use two sugarcane genotypes, YA05-179 (smut-resistant) and ROC22 (smut-susceptible), to identify differentially expressed miRNAs in sugarcane challenged with *S. scitamineum* by using high-throughput sequencing.

**Results:**

The predicted target gene number corresponding to known differentially expressed miRNAs in YA05-179 was less than that in ROC22, however most of them were in common. Expression of differential miRNAs under *S. scitamineum* challenge was mostly downregulated, with similar trends in the two varieties. Gene ontology (GO) analysis showed that the target gene classification of known miRNAs was similar to that of the newly identified miRNAs. These were mainly associated with cellular processes and metabolic processes in the biological process category, as well as combination and catalytic activity in the molecular function category. Kyoto Encyclopedia of Genes and Genomes (KEGG) pathway enrichment analysis revealed that these predicted target genes involved in a series of physiological and biochemical pathways or disease resistance-related physiological metabolism and signal transduction pathways, suggesting that the molecular interaction mechanism between sugarcane and *S. scitamineum* was a complex network system. These findings also showed certain predicted target genes of miR5671, miR5054, miR5783, miR5221, and miR6478 play roles in the mitogen-activated protein kinase (MAPK) signaling pathway, plant hormone signal transduction, and plant-pathogen interaction. Quantitative real-time PCR (qRT-PCR) analysis showed that majority of the known miRNAs and its predicted target genes followed a negatively regulated mode. Seven out of eight predicted target genes showed identical expression after 12 h treatment and reached the highest degree of matching at 48 h, indicating that the regulatory role of miRNAs on the target genes in sugarcane was maximized at 48 h after *S. scitamineum* challenge.

**Conclusions:**

Taken together, our findings serve as evidence for the association of miRNA expression with the molecular mechanism underlying the pathogenesis of sugarcane smut, particularly on the significance of miRNA levels in relation to the cultivation of smut-resistant sugarcane varieties.

**Electronic supplementary material:**

The online version of this article (doi:10.1186/s12864-017-3716-4) contains supplementary material, which is available to authorized users.

## Background

Sugarcane is the most important sugar crop in China, accounting for 92% of the total sugar production in the country. To date, sugarcane smut caused by *Sporisorium scitamineum* widely occurs in sugarcane fields worldwide and has become one of the most difficult fungal diseases to control [1, 2]. Due to poor resistance to sugarcane smut, several prevalent sugarcane varieties in China such as NCO310, F134, and CP73-351 have become vulnerable to smut disease, thereby resulting in major losses in cane yield and sugar and thereby have been eliminated in succession [2]. The cultivation of smut-resistant sugarcane varieties is considered as the most cost-effective approach in controlling this disease [3]. Hence, in-depth studies of disease resistance mechanisms are imperative for the prevention and control of sugarcane smut.

Extensive studies on the mechanism of interaction between sugarcane and *S. scitamineum* have been conducted in the past few years [4–16]. Heinzeetal et al. [4] used suppression subtractive hybridization (SSH) to obtain two full-length genes that were potentially important for the interaction between sugarcane and *S. scitamineum*, which in turn could facilitate in the evaluation of the two molecular markers for sugarcane smut resistant varieties. Borrás et al. [5] used cDNA-amplified fragment length polymorphism (cDNA-AFLP) technology to study the differential gene expression of sugarcane after the development of smut disease and screened 62 differentially expressed genes, including 52 upregulated genes and ten downregulated genes. Among these 52 upregulated genes, 19 were directly related to biological functions such as defense and signal transmission. Que et al. [6] used two-dimensional gel electrophoresis (2DE), matrix-assisted laser desorption/ionization time-of-flight mass spectrometry (MALDI-TOF MS), and quantitative PCR to comprehensively analyze the molecular responses, including transcription and protein expression in relation to the interaction between sugarcane and *S. scitamineum*. By RNA sequencing and isobaric tags for relative and absolute quantitation (iTRAQ), the gene and protein expression profiles of sugarcane in response to the infection of *S. scitamineum* were constructed, and its relationship with the mechanism of interaction between sugarcane and *S. scitamineum* was identified [7]. In 2015, Su et al. [8] developed a rapid and visual loop-mediated isothermal amplification (LAMP) for the detection of *S. scitamineum* in sugarcane. This assay was nearly 100 times more sensitive than conventional PCR for detection of this pathogen [8]. Su et al. also obtained three members of the β-1,3-glucanase gene family (*ScGluA1*, *ScGluD1* and *ScGluD*2) [9, 10], one catalase gene (*ScCAT1*) [11] and ten chitinase family genes [12], which are associated with the pathogenicity of sugarcane smut. Wu et al. [13] used Solexa high-throughput sequencing for differential gene expression profiling of sugarcane after infection with sugarcane smut and 2,015 differentially expressed sequence tags (ESTs) were screened, including three upregulated ESTs that were related to the mitogen-activated protein kinase (MAPK) signaling pathways. In addition, our group was the first to report the whole-genome sequence of *S. scitamineum* and to comprehensively describe the pathogenesis of sugarcane smut [14], and two other groups, Taniguti et al. [15] and Dutheil et al. [16], followed suit. It is the fourth smut fungal species subjected to whole-genome sequencing, following *Ustilago maydis* and *S. reilianum* in maize [17, 18] and *Ustilago hordei* in barley [19]. The aforementioned findings have promoted research on the molecular response involved in the interaction between sugarcane and smut fungus. However, no mechanistic analysis of microRNA (miRNA) differential expression and functional analysis of the potential target gene of sugarcane under *S. scitamineum* challenge has been conducted to date.

miRNAs are a class of non-coding small RNA (sRNA) of unequal lengths that ranging from 20 to 25 nt [20–22]. These have important biological functions, and its mediated post-transcriptional gene regulation is an extremely important sRNA regulatory pathway in in vivo models [20–22]. In 1993, Lee et al. [23] first described a miRNA in *Caenorhabditis elegans* by showing that an sRNA fragment that was complementary to the *lin-14* genomic sequence was a *lin-14* transcript, which could negatively regulate *lin-14* expression. In 2000, Reinhar et al. [24] found a gene, *let-7*, in *C. elegans* with a similar function as that of *lin-14*, further confirming that miRNA-mediated transcriptional regulation commonly but not coincidentally existed in organisms. Ruvkun et al. [25] in 2004 described a novel post-transcriptional gene expression regulatory mechanism that involved miRNA target genes. Since then, a large number of novel miRNAs have been reported in various plant and animal species, including human [26], mouse [27], *Drosophila* [28], nematodes, and *Arabidopsis* [29].

Previous studies have shown that miRNAs do not only influence the growth and development of organisms by regulating transcription factors, but also degrade target gene mRNAs or stop the target gene translation to change the cellular behavior of plants through numerous physiological pathways such as protein hydrolysis, metabolism, and ion transport [30], as well as signal transduction pathways [31–33]. Compared to miRNAs in animals, studies on plant miRNAs have lagged behind and only begun in 2002 [34]. Napoli et al. [35] confirmed the presence of miRNAs and small interfering RNAs (siRNAs), including its mechanism of interaction. They also explained the specific mechanism underlying co-suppression during the synthesis of flavonols and anthocyanins in plants [35]. A subsequent study showed that miRNAs played important biological functions in plants, wherein they are not only involved in various physiological and biochemical processes and regulate normal growth and development of organisms by controlling the expression of transcription factors [30–33], but also had a regulatory role in abiotic or biotic stresses [36, 37]. Jones et al. [38] showed that plants subjected to drought, cold, and high salinity stress induced changes in the expression of miR319c, miR393, miR395, miR397b, and miR402. For example, the ATP sulfurylase 1 gene (*APS1*), a target gene of miR395, significantly decreased under low sulfate stress. Patade et al. [39] revealed that sugarcane miR159 plays an important role in its response to high salinity stress. Lu et al. [40] obtained 48 miRNA sequences from poplars whose target genes were associated with growth and development, stress responses, anti-virus infection, and also other life-related processes. A previous study showed correlations between miRNA and virus-induced illness, as well as virus-mediated gene silencing [41]. Kasschau et al. [42] demonstrated that overexpression of the helper component-proteinase (*Hc-Pro*) gene in *Arabidopsis* plants significantly reduced miR171 expression and further increased target gene expression of miR171, thereby resulting in miR171-related developmental deficiency in plants.

This study focused on the interaction of two different sugarcane genotypes YA05-179 (smut resistant) and ROC22 (smut susceptible) inoculated with *S. scitamineum* 48 h by conducting differential miRNA expression analysis and quantitative real-time PCR (qRT-PCR) validation to identify and analyze the expression patterns of miRNA in sugarcane during *S. scitamineum* challenge, as well as functional analysis to predict its target genes. Moreover, the present study provides evidence on the role of miRNA against *S. scitamineum* challenge in sugarcane to further broaden and strengthen our understanding of the molecular mechanism underlying the response of sugarcane against smut disease, thereby providing a theoretical basis for the cultivation of smut-resistant sugarcane varieties.

## Methods

### Plant growth and stress treatment

Smut spores were collected from the host variety of ROC22, which were propagated at the Key Laboratory of Sugarcane Biology and Genetic Breeding, the Ministry of Agriculture/Fujian Agriculture and Forestry University (Fuzhou, China). The smut spores were placed in a paper bag, air-dried, and subsequently stored in a sealed container at 4 °C until use. The sugarcane varieties used in the experiments, ROC22 (smut-susceptible genotype) and YA05-179 (smut-resistant genotype), were provided by the Key Laboratory of Sugarcane Biology and Genetic Breeding, Ministry of Agriculture/Fujian Agriculture and Forestry University. Healthy and uniform sugarcane (from the 4^th^ to 7^th^ nodes counting from the basal node) were collected and cut into single-bud setts, followed by soaking in clean running water for 1 d. The treated materials were then incubated at 28 °C and cultivated in a moisturizer until the sugarcane buds grew to 1–2-cm in length, followed by needle puncture inoculation of smut spores suspension (5 × 10^6^ spores/mL, with 0.01% volume ratio of Tween-20) into the sugarcane buds. The control groups were injected with sterile water (with 0.01% volume ratio of Tween-20). After treatment, the sugarcane buds of the two groups were continuously incubated at 28 °C with conditions of 12 h light and 12 h dark photoperiods [8]. To minimize biological variance, three sugarcane buds were collected at each time point (i.e., 0, 12, 48, and 96 h) after inoculation and mixed well, followed by snap freezing in liquid nitrogen and storing at −80 °C until use.

### RNA isolation and sequencing

Total RNA was extracted from the ROC22 and YA05-179 sugarcanes in the treated and control groups using TRIzol^TM^ (Invitrogen, Carlsbad, CA, USA). RNA integrity was determined by 1% agarose gel electrophoresis. The concentration of total RNA was determined by using an Agilent Bio-analyzer 2100 system (Agilent Technologies, Santa Clara, CA, USA). The samples of ROC22 and YA05-179 inoculated with sterile water and smut spores for 48 h, namely, RCK (control group of ROC 22), RT (treatment group of ROC22), YACK (control group of YA05-179), and YAT (treatment group of YA05-179) were used for the construction of sRNA libraries (Beijing Genomics Institute, Beijing, China). sRNA fragments (18–30 nt) collected from 15% PAGE were each connected to 5′ end and 3′ end adapters, followed by reverse transcription and PCR analysis and were then sequenced using a HiSeq2000 sequencing system.

### Sequencing data processing and analysis

The adapters at both ends of the 50-nt fragments obtained from the high-throughput sequencing were detached, followed by removal of contaminating sequences and low-quality reads to obtain clean reads. Then, statistical analysis of sequence length distribution, as well as common and specific sequences among samples was performed. The length of sRNA ranged from 18 to 30 nt, with miRNAs normally 21–22 nt in length, siRNA 21–24 nt, and Piwi-interacting RNA (piRNA) within the range of 28–30 nt [43]. The peak value representing the quantity of different lengths of sRNAs was either 21 nt or 24 nt in length for plant samples, whereas the peak value of sRNAs of animal samples was 22 nt [43]. Classification and annotation of clean sequences were performed to generate information on each component and expression levels of each sample. Using GenBank (http://www.ncbi.nlm.nih.gov/) and Rfam 10.1 (http://rfam.sanger.ac.uk) databases comparison, non-coding RNAs such as ribosomal RNA (rRNA), transfer RNA (tRNA), small nuclear RNA (snRNA), snoRNA, and signal recognition particle RNA (srpRNA) were identified and removed. The remaining sRNAs were subjected to a Basic Local Alignment Search Tool (BLASTn) search with no more than two mismatches against miRBase 18.0 database (http://www.mirbase.org/) to identify known miRNA in the samples [44, 45]. sRNA annotation followed a priority rule for classification to avoid redundancy: non-coding RNA (in which GenBank > Rfam) > known miRNA > repeat [46]. Due to the lack of genome information for sugarcane, the sequences that did not match known miRNAs were mapped to the Sugarcane_Unigene database (65,852 unigenes) established by our previous transcriptome analysis in ROC22 and YA05-179 post-*S. scitamineum* infection for 24, 48 and 120 h [47] and the sugarcane EST in GenBank and the *S. scitamineum* genome database [14] to identify potentially novel miRNA candidates. Mireap (http://sourceforge.net/projects/mireap/) with default parameters was used to predict the sRNAs without annotation and prepare the secondary structure of the novel miRNA.

### Differential expression of *S. scitamineum*-responsive miRNAs

To understand the differential expression of sugarcane miRNA after *S. scitamineum* challenge, the observed frequencies of unique sequences were normalized to the reads per million (RPM) data. If the original miRNA expression in a library was zero, the normalized read count of this miRNA was adjusted to 0.01 in the library for further calculation [48–50]. We performed statistical analysis of known miRNAs, as well as novel miRNAs to identify significant differences in the expression between the treatment and control groups (comparisons: RT vs. RCK or YAT vs. YACK). Then we used the graphs of log_2_-ratio and scatterplot to compare the expression level of miRNAs expressed by both groups. The specific procedures are as follows: (1) treatment and control groups were normalized to the same orders of magnitude. Formula: Normalized expression level = miRNA expression level/total expression level of the sample × normalized magnitude; (2) Normalized results were used to calculate the fold change and *P*-value, as well as for graph preparation. The formula for calculating fold change was as follows: Fold change = log_2_ (treatment vs. control). The *P*-value was calculated based on the following equation [51]:

$$ p\left( y\left| x\right.\right)={\left(\frac{N2}{N1}\right)}^y\frac{\left( x+ y\right)!}{x! y!{\left(1+\frac{N2}{N1}\right)}^{\left( x+ y+1\right)}}\kern1.5em \begin{array}{c}\hfill C\left( y\le {y}_{\min}\left| x\right.\right)={\displaystyle \sum_{y=0}^{y\le {y}_{\min }} p\left( y\left| x\right.\right)}\hfill \\ {}\hfill D\left( y\ge {y}_{\max}\left| x\right.\right)={\displaystyle \sum_{y\ge {y}_{\max}}^{\infty } p\left( y\left| x\right.\right)}\hfill \end{array} $$

where *x* means control, *y* means treatment, *N*_*1*_ means the normalized expression of a miRNA in the control library, and *N*_*2*_ means the normalized expression of the same miRNA in the treatment library. C and D are used to estimate the confidence intervals [*y*_min_, *y*_max_] in regards to a specific *P*-value.

The value of fold change >1 or < −1 and *P*-value <0.05 were used as criteria in screening for miRNAs that were significantly differentially expressed between samples. In addition, the present study performed cluster analysis of differentially expressed miRNAs in the two sugarcane varieties, i.e., cluster analysis was performed to identify differentially expressed known and novel miRNAs in the treated sugarcane samples after *S. scitamineum* challenge and the control samples inoculated with sterile water [52].

### miRNA target gene prediction and functional analysis

With reference to the Sugarcane_Unigene database (65,852 unigenes) [47] and the sugarcane EST in GenBank, the present study used the psRNATarget online software (http://plantgrn.noble.org/psRNATarget/) to predict the target genes of the known miRNAs and the novel miRNAs. The specific prediction standards were based on Allen et al. [53] and Schwab et al. [54]. Then, these predicted target genes of differentially expressed miRNAs in RT/RCK and YAT/YACK were subjected to Gene Ontology (GO, http://www.geneontology.org/) enrichment and Kyoto Encyclopedia of Genes and Genomes (KEGG, http://www.genome.jp/kegg/) pathway analyses [55].

### Validation of miRNAs and its predicted target genes by qRT-PCR analysis

qRT-PCR was performed to determine the expression levels of 20 differentially expressed miRNAs. These 20 miRNAs included 16 known miRNAs and 4 novel miRNAs. Primer 5.0 software was used to design upstream primers (Additional file 1: Table S1), whereas downstream primers were derived from the Uni-miR qPCR Kit purchased from Takara (Dalian, China). The internal reference was *5S rRNA* [56]. The sugarcane buds from ROC22 and YA05-179 inoculated with distilled water and *S. scitamineum* at 0 and 48 h were used for qRT-PCR samples. Reverse transcription was conducted using the One Step PrimeScript® miRNA cDNA Synthesis Kit (Perfect Real Time) (Takara, China), following the manufacturer’s instructions. Polyadenylation reaction was used to detect miRNA expression. The 2^−ΔΔCt^ method [57] was used to calculate the miRNA expression levels of ROC22 and YA05-179 at 48 h after *S. scitamineum* infection. Moreover, qRT-PCR was used to determine the expression patterns of the 12 miRNAs in ROC22 and YA05-179 at 0, 12, 48, and 96 h after *S. scitamineum* challenge.

Beacon Designer 7.0 software was used to design the quantification primers of the 23 randomly selected miRNA target genes (Additional file 2: Table S2). qRT-PCR was used to analyze the expression patterns of the predicted target genes in ROC22 and YA05-179 at 0, 12, 48, and 96 h after *S. scitamineum* challenge. The glyceraldehyde-3-phosphate dehydrogenase (*GAPDH*) gene was used as internal reference [58]. Reverse transcription was performed using the PrimeScript^TM^ RT-PCR Kit (Takara, Dalian, China), following the manufacturer’s instructions. For qRT-PCR expression analysis, the total volume of reaction system was 20 μL, which included 10 μL SYBR Premix *Ex Taq* ^TM^ II (2×) (Takara), 0.8 μL each of the upstream and downstream primers (10 μM), 1 μL of the cDNA template, and 7.4 μL of ddH_2_O. The reaction conditions were 50 °C for 2 min and 95 °C for 60 s; followed by 40 cycles of 95 °C for 15 s and 60 °C for 60 s; and 95 °C for 15 s; 60 °C for 60 s; 95 °C for 15 s. All assays were performed in triplicate. At the end of amplification, the 2^−ΔΔCt^ method [57] was used to calculate the results of qRT-PCR analysis. Statistical analysis was conducted using the Data Processing System (DPS) v7.05 software (China). Data were expressed as the mean ± standard error (SE). Significance (*P*-value <0.05) was calculated using one-way Analysis of Variance (ANOVA) followed by Duncan’s new multiple range test.

## Results

### Categories and size distribution of sRNAs in sugarcane after challenging with *S. scitamineum*

Sequence impurities generated from sRNA sequencing generally refers to contaminated sequences such as non-insert fragments, fragments without 3′ adapters or fragments with only 5′ adapters, reads containing polyA sequences, and sequences with lengths <18 nt [59]. In the present study, the four samples, RCK, RT, YACK, and YAT, underwent sequencing and all obtained 3 × 10^8^ high-quality 18–30 nt clean reads, in which 36,396,588, 27,812,972, 27,464,468, and 28,290,231 reads were from each library, respectively (Additional file 3: Table S3). Additional file 4: Figure S1 shows that the tested sRNA sequence length of the four samples were mainly within the range of 20–24 nt, a typical size of Dicer-derived sRNAs, in which, majority of the reads were 24 nt in length, and accounted for 45.09, 54.26, 55.80, and 59.24% of the read in RACK, RT, YACK, and YAT, respectively. The proportions of 20–22 nt sRNAs in the treated sugarcane samples of ROC22 and YA05-179 after *S. scitamineum* challenge were relatively less than that observed in the controls, whereas the proportion of 23 nt–24 nt sRNAs in the treated sugarcane samples of ROC22 and YA05-179 after *S. scitamineum* challenge were relatively higher than that of the controls. These findings indicated that *S. scitamineum* induces changes in the expression pattern of sRNAs in both sugarcane genotypes.

**Fig. 1:**
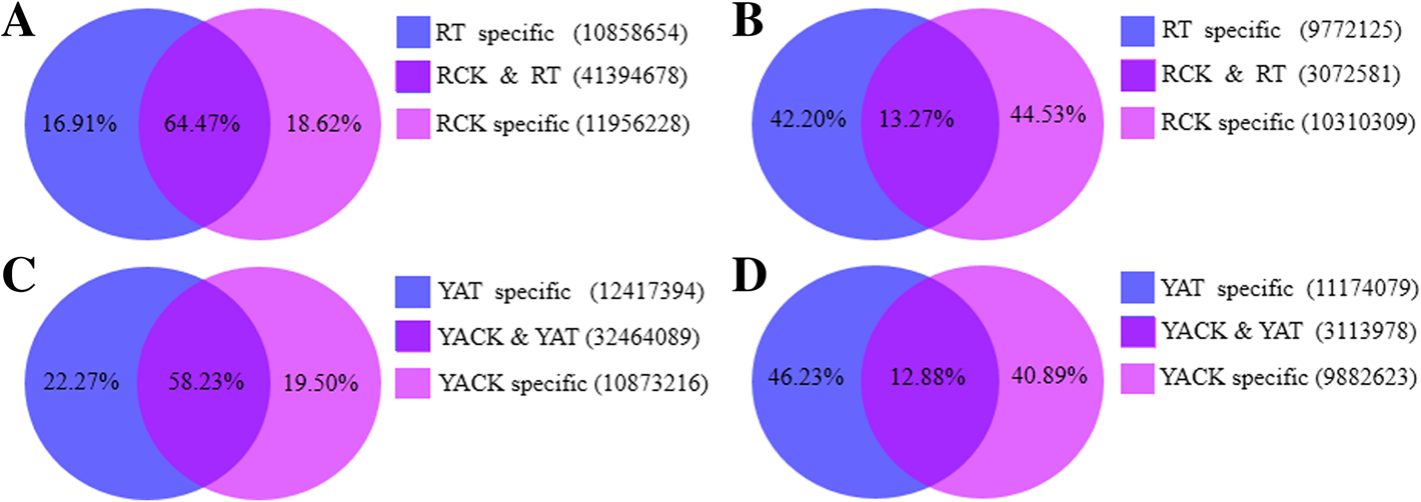
The common and specific sRNAs in ROC22 and YA05-179 after *Sporisorium scitamineum* challenge. **a** Summary of total sRNAs between RT and RCK. **b** Summary of unique sRNAs between RT and RCK. **c** Summary of total sRNAs between YAT and YACK. **d** Summary of unique sRNAs between YAT and YACK. RCK and YACK: ROC22 and YA05-179 under sterile water stress after 48 h, respectively; RT and YAT: ROC22 and YA05-179 under *S. scitamineum* stress after 48 h, respectively

Comparison of the sRNA libraries of the *S. scitamineum*-treated and the control groups (RT vs. RCK and YAT vs. YACK) indicated that only less than 15% of the unique reads were shared by the two sRNA libraries. Figure 1a and b show that the common sequences between RT and RCK accounted for 64.47% of the total reads and 13.27% of the unique reads, respectively. Figure 1c and d show that the common sequences between YAT and YACK accounted for 58.23% of the total reads and 12.88% of the unique reads. The observation of unique reads with broad specificity suggests that these may be related to the resistant phenotype of sugarcane to *S. scitamineum*.

The sRNAs obtained in the present study were concentrated within the sense and antisense strands of rRNA and sense strand of tRNA (Additional file 5: Table S4). Matching of the sRNA sequences of small cytoplasmic RNA (scRNA), rRNA, snRNA, small nucleolar RNA (snoRNA), and tRNA obtained from the GenBank and Rfam 10.1 databases were then performed (Additional file 6: Table S5 and Additional file 7: Table S6). Table 1 shows the annotated of sRNAs. Among the unique sequences, 62,110 (0.46%), 56,796 (0.44%), 58,020 (0.45%), and 60,639 (0.42%) were identified to be similar to known miRNAs in RCK, RT, YACK, and YAT after searching the miRBase, respectively. Other types of unique sequences, including rRNA, snRNA, snoRNA, srpRNA, and tRNA were also detected in the four libraries. However, there were 13,132,245 (98.13%), 12,686,933 (98.77%), 12,816,045 (98.61%), and 14,130,110 (98.89%) unique sequences in RCK, RT, YACK, and YAT that could not be annotated, respectively, suggesting these sRNAs may be unique to sugarcane.

**Table 1:** Annotation and classification of the small RNAs in the four libraries

Category	RCK		RT		YACK		YAT	
	Unique	Total	Unique	Total	Unique	Total	Unique	Total
Total reads	13,382,890	36,396,588	12,844,706	27,812,972	12,996,601	27,464,468	14,288,057	28,290,231
miRNA reads	62,110	3,842,212	56,796	2,769,304	58,020	2,117,246	60,639	1,973,307
rRNA reads	149,095	4,535,331	80,458	1,347,015	97,711	1,924,796	76,758	1,102,526
repeat reads	526	1,214	489	1,114	424	1,067	443	1,153
snRNA reads	6,390	25,149	4,072	11,579	4,800	12,774	4,897	12,309
snoRNA reads	4,381	13,333	2,596	5,793	2,676	6,200	2,545	5,375
srpRNA reads	2	2	0	0	0	0	0	0
tRNA reads	28,141	1,578,262	13,362	475,165	16,925	588,689	12,665	323,280
Unannotaed sRNA reads	13,132,245	26,401,085	12,686,933	23,203,004	12,816,045	22,813,696	14,130,110	24,870,281

RCK and YACK: ROC22 and YA05-179 under sterile water stress after 48 h, respectively; RT and YAT: ROC22 and YA05-179 under *Sporisorium scitamineum* stress after 48 h, respectively

### Identification of known and novel miRNAs

miRNA prediction was performed according to the formation and biometrics of miRNAs [60]. In the present study, the sequences of the miRNA precursors and mature miRNA in the four sugarcane samples in the unique sRNAs and miRBase databases were compared. BLAST analysis identified a total of 264, 263, 260, and 262 known miRNAs in RCK, RT, YACK, and YAT, respectively. The Mireap software was used to compare and screen the unannotated sequences (unann) using sugarcane ESTs from GenBank and transcriptome database of sugarcane in response to smut infection (65,852 unigenes) to predict novel miRNAs. Our results showed that there were 137, 140, 111, and 119 novel miRNAs in RCK, RT, YACK, and YAT, respectively. All of the miRNAs obtained by sequencing were mapped to the *S. scitamineum* genome database reported by Que et al. [14]. The result showed that none of the miRNA sequences was mapped to *S. scitamineum* genome sequence, suggesting there was no contaminated *S. scitamineum* reads in the identified sugarcane miRNAs. Additional file 8: Figure S2A shows that the base distribution of the leading sites of novel miRNAs in the four samples were very similar. For example, the leading base of novel miRNAs of 20 nt in length was C, and majority of the leading base of novel miRNAs of 21 and 22 nt lengths was U. The distribution and proportion of the four bases in the leading sites of novel miRNAs of 23 nt in length were similar. The base distribution of novel miRNAs at different sites was identical in the four samples (Additional file 8: Figure S2B). In addition, the 11th nucleotide of the candidate miRNA sequences from all samples was generally A.

### *S. scitamineum*-responsive miRNAs

**Fig. 2:**
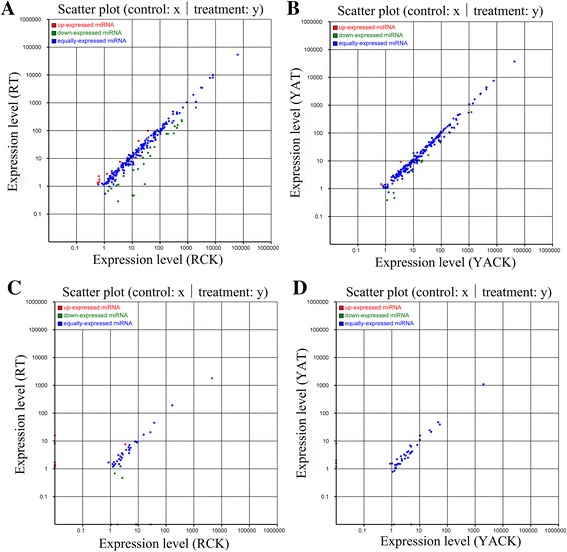
Expression of miRNAs in ROC22 and YA05-179 after *Sporisorium scitamineum* challenge. **a** The differences of known miRNAs expression between RT and RCK. **b** The differences of known miRNAs expression between YAT and YACK. **c** The differences of novel miRNAs expression between RT and RCK. **d** The differences of novel miRNAs expression between YAT and YACK. The scatter plot of differentially expressed miRNAs (control: X-axis, treatment: Y-axis). The X and Y show the expression level of miRNAs in the two strains respectively. *Red points* mean miRNAs with log_2_-ratio >1; *Blue points* mean miRNAs with −1 ≤ log_2_-ratio ≤1; *Green points* mean miRNAs with log_2_-ratio <1. Ratio = normalized expression of the treatment/normalized expression of the control. RCK and YACK: ROC22 and YA05-179 under sterile water stress after 48 h, respectively; RT and YAT: ROC22 and YA05-179 under *S. scitamineum* stress after 48 h, respectively

Transcriptional regulation in plants mainly relies on differentially expressed miRNAs [61]. In the present study, scatter plot analysis (Fig. 2a and b) showed that most differentially expressed known miRNAs in ROC22 and YA05-179 after *S. scitamineum* challenge was equally-expressed miRNAs. The number of upregulated miRNAs was less than that of downregulated miRNAs. Under the screening criteria of log_2_-ratio >1 or < −1 and *P*-value <0.01, nine significantly upregulated and 26 significantly downregulated miRNAs (Additional file 9: Table S7) were detected among the 231 differentially expressed known miRNAs in the RT/RCK comparison; and nine significantly upregulated and two significantly downregulated miRNAs (Additional file 10: Table S8) were observed among the 208 differentially expressed known miRNAs in the YAT/YACK comparison. The value of differential fluctuation of differentially expressed known miRNAs in ROC22 and YA05-179 was up to −4.61- (miR5242) and −2.15-fold (miR5152-3p), respectively.

The number of differentially expressed novel miRNAs was lower than that of known differentially expressed miRNAs. Most of the differentially expressed novel miRNAs were equally expressed miRNAs (Fig. 2c and d). RT/RCK comparison identified nine significantly upregulated and eight significantly downregulated miRNAs (*P*-value <0.01) among the 48 differentially expressed novel miRNAs (Additional file 11: Table S9). In the YAT/YACK comparison, two significantly upregulated and four significantly downregulated miRNAs (*P*-value <0.01) were detected among the 41 differentially expressed novel miRNAs (Additional file 12: Table S10). Interestingly, the differentially expressed novel miRNAs with highly significant differential expression levels (*P*-value <0.01) were specifically expressed in both sugarcane varieties. The differential fluctuation of differentially expressed novel miRNAs in ROC22 and YA05-179 was up to −11.46- (novel_mir_11) and 9.65-fold (novel_mir_187), respectively.

**Fig. 3:**
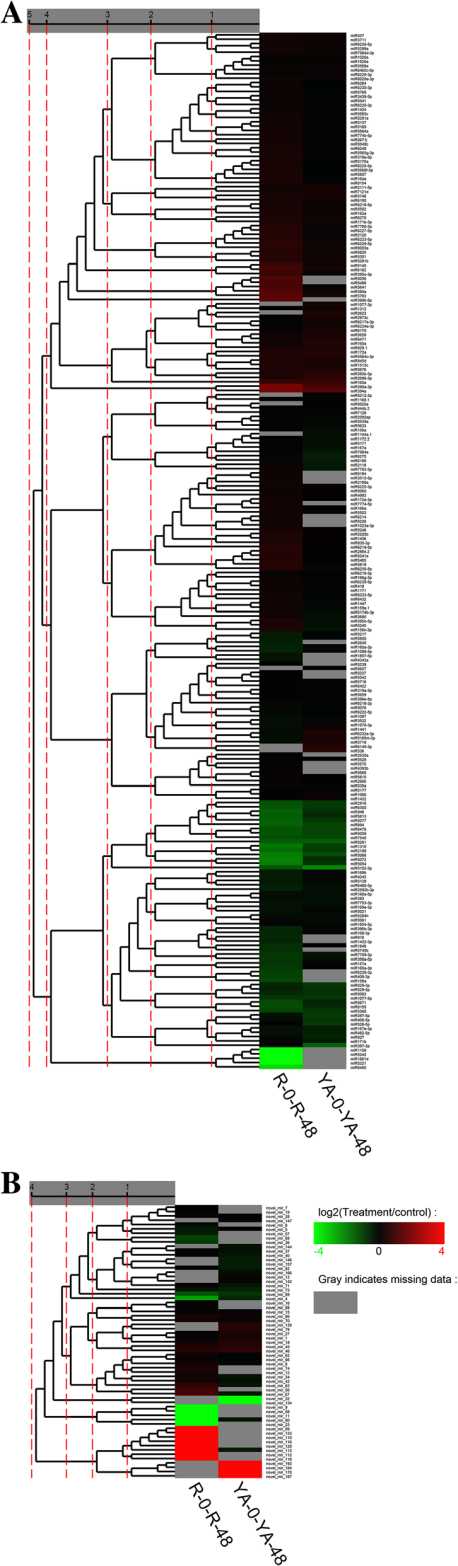
Hierarchical clustering of differentially expressed known (**a**) and novel (**b**) miRNAs in ROC22 and YA05-179 after *Sporisorium scitamineum* challenge. R-0 and YA-0: ROC22 and YA05-179 under sterile water stress after 48 h, respectively; R-48 and YA-48: ROC22 and YA05-179 under *S. scitamineum* stress after 48 h, respectively

Although differences in miRNA expression levels were significant, the expression trends (either up- or downregulated) of differentially expressed known miRNAs in both sugarcane varieties (RT/RCK and YAT/YACK) were generally similar (Fig. 3a). The specific regulatory modes of differentially expressed known miRNAs could be divided into four categories: significantly upregulated, upregulated in trace amounts, downregulated in trace amounts, and significantly downregulated. Most of the differentially expressed known miRNAs were categorized as significantly downregulated. Data analysis showed that the eight known miRNAs, miR1077-3p, miR262, miR5212-3p, miR5020a, miR1144a.1, miR5657, miR6149-3p, and miR536 were specifically expressed in YA05-179, but not in ROC22. Figure 3b shows that differentially expressed novel miRNAs were mostly detected in a single variety of sugarcane, but not in both sugarcane varieties.

### Prediction of target genes of miRNAs in sugarcane

In the present study, the targeted genes of the known miRNAs, and novel miRNAs were subjected to prediction. The results indicated that most of the miRNAs had approximately ten target genes. Some miRNAs even had >100 target genes. According to the results of target gene prediction of known miRNAs (Additional file 13: Table S11), 32 differentially expressed known miRNAs of RT/RCK had 814 target genes, and ten differentially expressed known miRNAs of YAT/YACK had 127 target genes. Target gene prediction of novel miRNAs (Additional file 14: Table S12) showed that the 15 differentially expressed miRNAs of RT/RCK had 457 target genes, and the six differentially expressed miRNAs of YAT/YACK were associated with 1,754 target genes. Additional file 14: Table S12 presents the prediction results for partial target genes of differentially expressed miRNAs. Analysis indicated that the function of these unannotated miRNA target genes were unknown or poorly characterized, thereby suggesting the possibility of new roles for these miRNAs in sugarcane in response to *S. scitamineum*.

### GO analysis of the predicted target genes

GO enrichment analysis was conducted for the predicted target genes of differentially expressed miRNAs in RT/RCK and YAT/YACK. Additional file 15: Figure S3 and Additional file 16: Figure S4 demonstrate the GO classification of the predicted target genes of known and novel miRNAs associated with biological processes, cellular components, and molecular functions. The main GO classification of target genes of differentially expressed known miRNAs and novel miRNAs in YAT/YACK and RT/RCK was similar, which demonstrated that the differentially expressed miRNAs of the two sugarcane varieties after *S. scitamineum* challenge mainly targeted genes that were associated with cellular processes and metabolic processes. The predicted target genes were mainly associated with cell and organelle components. The molecular functions of the predicted target genes were mainly related to binding and catalytic activities.

### KEGG analysis of the predicted target genes

KEGG pathway enrichment analysis identifies the most important physiological metabolic pathways and signal transduction pathways of candidate target genes [12]. In RT/RCK, the predicted target genes of known miRNAs had 13 pathways that were enriched (*P* <0.05), and ten pathways that were significantly enriched (*P* <0.01) (Additional file 17: Table S13). In YAT/YACK, the predicted target genes of known miRNAs had four pathways that were enriched (*P* <0.05) and three pathways that were significantly enriched (*P* <0.01) (Additional file 18: Table S14), including pathogenic *Escherichia coli* infection, phagosome, and other types of O-glycan biosynthesis. In RT/RCK, the predicted target genes of novel miRNAs had 13 pathways that were enriched (*P* <0.05) and 11 pathways that were significantly enriched (*P* <0.01) (Additional file 19: Table S15). In YAT/YACK, the predicted target genes of novel miRNAs showed 13 pathways that were enriched (*P* <0.05) and five pathways that were significantly enriched (*P* <0.01) (Additional file 20: Table S16).

**Fig. 4:**
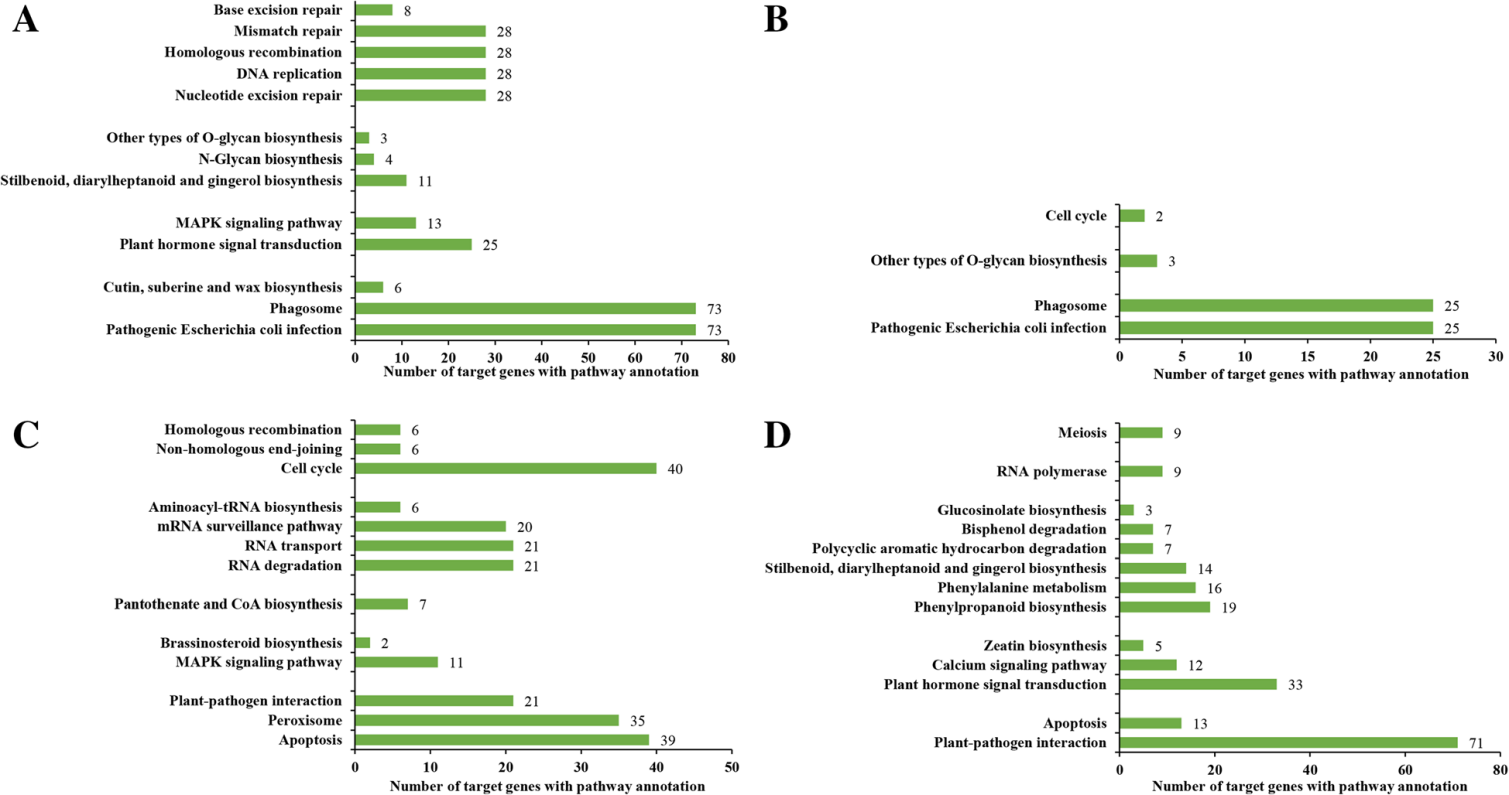
The significantly enriched KEGG pathway (*P*-value <0.05) of the predicted target genes. The genes were targeted by differently expressed known and novel miRNAs in RT/RCK (**a** and **c**) and YAT/YACK (**b** and **d**), respectively. RCK and YACK: ROC22 and YA05-179 under sterile water stress after 48 h, respectively; RT and YAT: ROC22 and YA05-179 under *S. scitamineum* stress after 48 h, respectively. Number of target genes with pathway annotation was shown in the bar charts

The significantly enriched KEGG pathway of the predicted target genes in sugarcane after *S. scitamineum* challenge (Fig. 4) could be divided into five types, including stress response pathway (i.e., plant-pathogen interaction, apoptosis, pathogenic *Escherichia coli* infection, phagosome, cutin, suberine and wax biosynthesis, and peroxisome), hormone and signal transduction pathways (i.e., calcium signaling pathway, MAPK signaling pathway, plant hormone signal transduction, zeatin biosynthesis, and brassinosteroid biosynthesis), metabolic pathway (i.e., stilbenoid, diarylheptanoid and gingerol biosynthesis, polycyclic aromatic hydrocarbon degradation, phenylalanine metabolism, bisphenol degradation, glucosinolate biosynthesis, other types of O-glycan biosynthesis, N-Glycan biosynthesis, and pantothenate and CoA biosynthesis), transcription and protein synthesis pathways (i.e., RNA polymerase, RNA degradation, mRNA surveillance pathway, RNA transport, and aminoacyl-tRNA biosynthesis), and cell division pathway (i.e., cell cycle, DNA replication, meiosis, nucleotide excision repair, homologous recombination, mismatch repair, base excision repair, and non-homologous end-joining). In general, miRNA target gene enriched pathways of both sugarcane varieties were mostly identical, but not in the number of target genes and the degree of significant enrichment in the pathways.

### Validation of miRNAs by qRT-PCR analysis

**Fig. 5:**
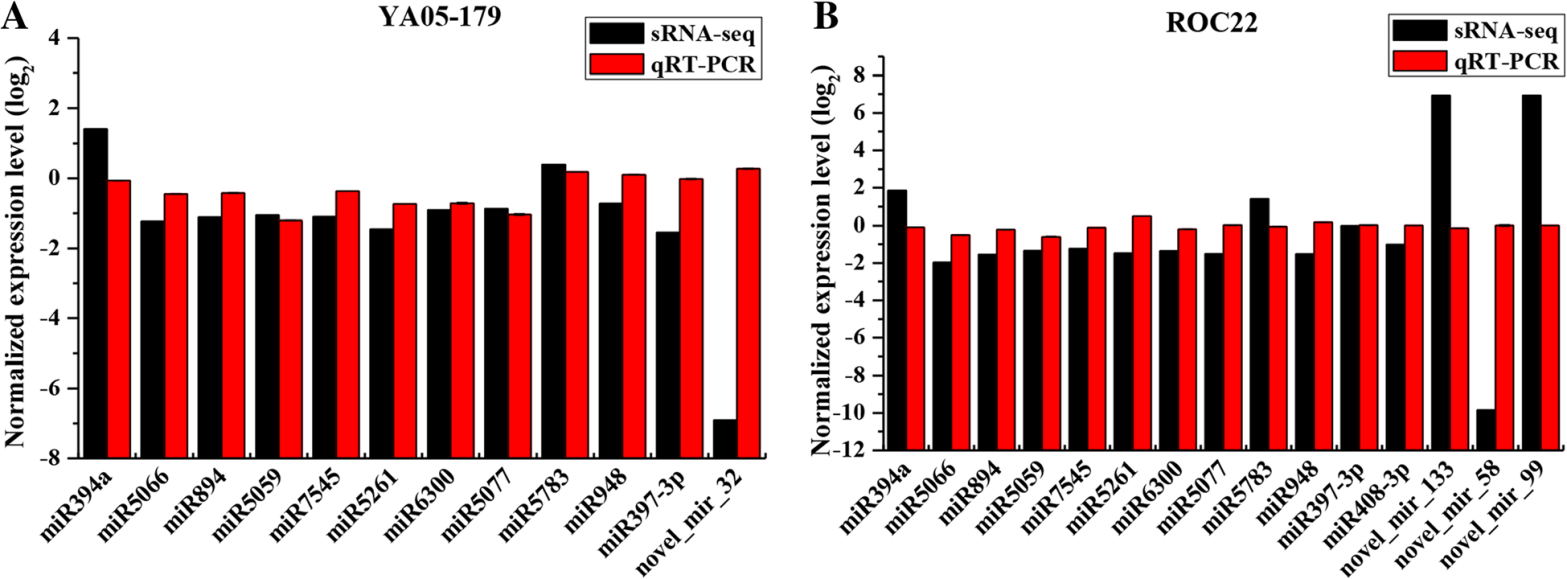
qRT-PCR validation of 16 randomly selected differentially expressed miRNAs identified by small RNA sequencing. **a** miRNAs expressed in YA05-17. **b** miRNAs expressed in ROC22. Sugarcane buds of ROC22 and YA05-179 post inoculation with sterile water or *Sporisorium scitamineum* for 48 h were used as qRT-PCR samples. The data of qRT-PCR were normalized to the *5S rRNA* expression level and represented as means of three replicates (*n* = 3) ± standard error

In the present study, 12 candidate differentially expressed known miRNAs with different expression levels, including miR394a, miR408-3p, miR397-3p, miR7545, miR5066, miR5261, miR948, miR5059, miR5783, miR5077, miR6300, and miR894, as well as four candidate differentially expressed novel miRNAs, including novel_mir_133, novel_mir_99, novel_mir_32, and novel_mir_58 were screened and further analyzed via qRT-PCR to verify their expression. Among the 16 miRNAs, miR408-3p, novel_mir_133, novel_mir_99, novel_mir_32, and novel_mir_58 were specifically expressed in a single sugarcane variety. As shown in Fig. 5, except for four miRNAs, including miR5261 and miR397-3p in YA05-179, as well as miR5077 and novel_mir_99 in ROC22, the miRNA expression patterns (up- or downregulated) as measured by quantitative analysis and the sequencing results of the miRNAs were generally similar.

**Fig. 6:**
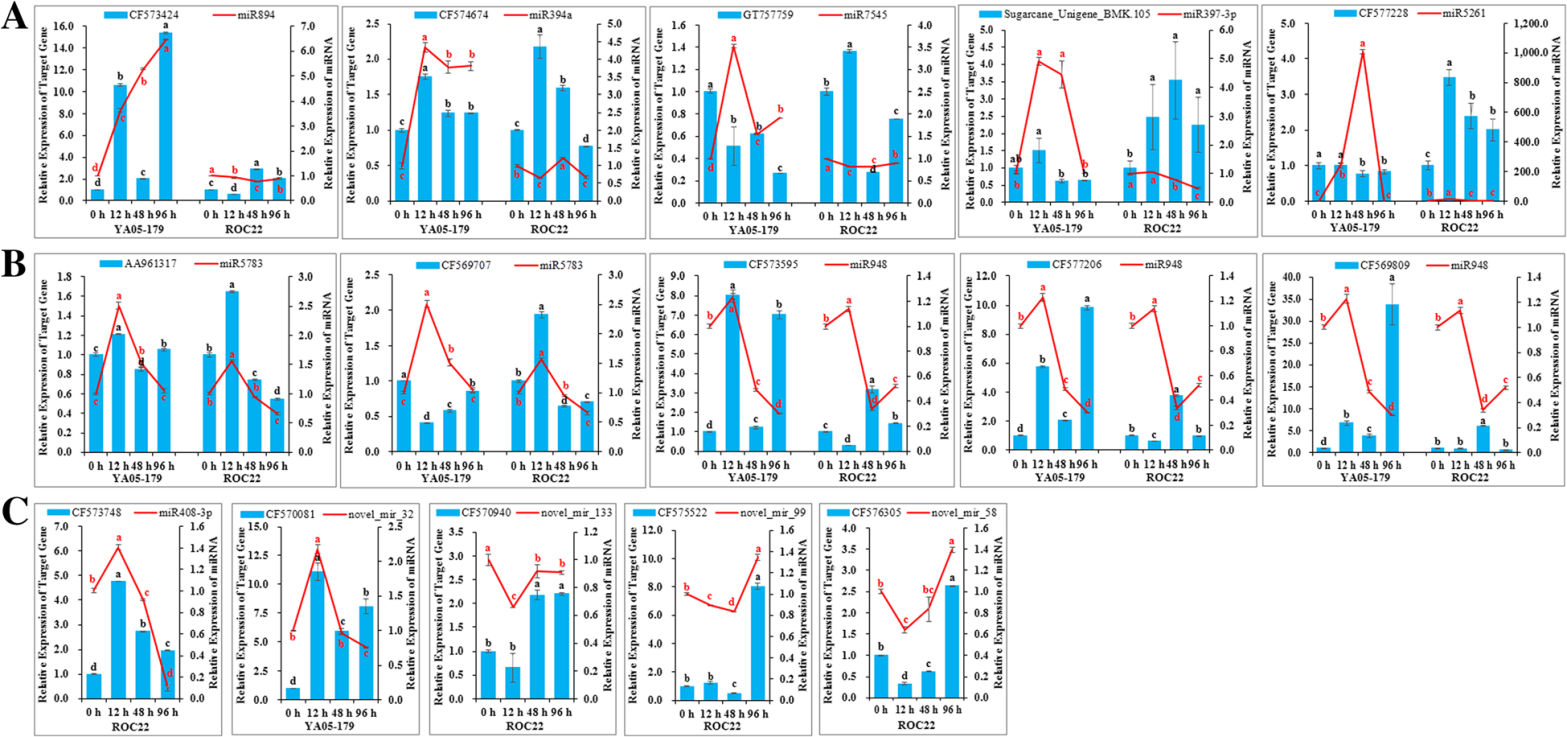
Expression patterns of selected 12 miRNAs (*line charts*) and 15 predicted target genes (*bar charts*) in ROC22 and YA05-179 at 0, 12, 48, and 96 h after *Sporisorium scitamineum* challenge. Gene expression levels were assessed by qRT-PCR. The *right and left y axis* represented the relative expression of miRNA and its predicted target gene, respectively. The relative expression levels of miRNAs and their predicted target genes were normalized to the *5S rRNA* and glyceraldehyde-3-phosphate dehydrogenase (*GAPDH*) expression levels, respectively. Each bar represented as means of three replicates (*n* = 3) ± standard error. Different lowercase letters indicate a significant difference, as determined by Duncan’s new multiple range test (*P*-value <0.05). The names of miRNA and corresponding targeted gene were listed in the top of each panel. Five out of twelve miRNAs, including miR408-3p, novel_mir_32, novel_mir_133, novel_mir_99, and novel_mir_58, were expressed specifically in ROC22 or YA05-179 after *S. scitamineum* challenge

In addition, we performed qRT-PCR analysis of the 12 candidate differentially expressed miRNAs at different time points (0, 12, 48, and 96 h) after *S. scitamineum* challenge. As shown in the line charts of Fig. 6, seven miRNAs, including miR394a, miR7545, miR894, miR397-3p, miR5261, miR5783, and miR948 were determined to be expressed in both sugarcane varieties, YA05-179 and ROC22. A specific expression pattern for miR5261 was observed, which involved a drastic upregulation in the sugarcane smut-resistant genotype, YA05-179, after inoculation of *S. scitamineum.* Its expression reached 1,000-fold at 48 h after *S. scitamineum* challenge and declined to 0-fold at 96 h. On the other hand, in the smut-susceptible variety, ROC22, miR5261 expression drastically increased after *S. scitamineum* challenge and reached 11-fold at 12 h post-inoculation, followed by a decline at 48 h and remained unchanged at 96 h. Among the seven miRNAs, the expression patterns of six miRNAs, except for miR5261, could be generally divided into two types: first, opposite expression patterns in YA05-179 and ROC22 (Fig. 6a), and second, identical expression pattern in YA05-179 and ROC22, which referred to similar relative expression regulatory trends of miRNAs in the two sugarcane varieties and at different treatment time points after *S. scitamineum* challenge (Fig. 6b). There were four miRNAs, which included miR394a, miR894, miR7545, and miR397-3p that demonstrated the first type of expression pattern, whereas miRNAs involved in the second expression pattern consisted of miR948 and miR5783. However, miR5783 was upregulated in YA05-179, but upregulated and subsequently downregulated in ROC22. On the other hand, both miR948 and miR5783 were upregulated within 12 h after *S. scitamineum* challenge, which was followed by its downregulation. In addition, the remaining five miRNAs (i.e., miR408-3p, novel_mir_133, novel_mir_99, novel_mir_58, and novel_mir_32) were only expressed in a single variety (Fig. 6c). Among these, miR408-3p, novel_mir_133, novel_mir_99, and novel_mir_58 were specifically expressed in ROC22. miR408-3p was markedly increased at 12 h and subsequently downregulated from 48 to 96 h after *S. scitamineum* challenge. Novel_mir_133 was downregulated at 12, 48, and 96 h compared to control. Novel_mir_99 and novel_mir_58 showing an overall downregulation, followed by a slight upregulation at 12 and 96 h. Novel_mir_32 in YA05-179, demonstrated an “upregulated-downregulated” specific expression pattern.

### Validation of the predicted target genes by qRT-PCR analysis

qRT-PCR analysis of 15 predicted target genes of differentially expressed known and novel miRNAs in different sugarcane varieties was conducted (Fig. 6) at different time points (i.e., 0–96 h) after *S. scitamineum* challenge. Despite the specific expression of predicted target gene CF573748 of differentially expressed known miRNA miR408-3p (Fig. 6c), the predicted target genes (i.e., CF570081, CF570940, CF575522, and CF576305) of four novel miRNAs (Fig. 6c) were detected in a single sugarcane variety, and the remaining predicted target genes were expressed in both sugarcane varieties (Fig. 6a and b).

Figure 6 shows the results of 11 predicted target genes of eight candidate differentially expressed known miRNAs. The expression of predicted target gene CF573748 in ROC22 was generally upregulated. The other predicted target genes were divided into two categories based on its expression patterns: (i) Significantly upregulated expression in ROC22, which was mostly higher than that in the upregulated expression in YA05-179. The observed upregulation in YA05-179 was gradual or mostly downregulated at 12 h after *S. scitamineum* challenge compared to that at 0 h of *S. scitamineum* challenge. This category included CF574674, Sugarcane_Unigene_BMK.105, CF577228, AA961317, CF569707, and GT757759. (ii) Significantly and generally upregulated expression in YA05-179 compared to that at 0 h of *S. scitamineum* challenge, whereas it was first downregulated, followed by moderate upregulation in ROC22 after *S. scitamineum* challenge. This category included CF573424, CF573595, CF577206, and CF569809. Figure 6c shows that the overall expression of the predicted target gene, CF570081, of novel miRNA novel_mir_32 in YA05-179 was upregulated, whereas that of the three predicted target genes, i.e., CF570940, CF575522, and CF576305 in ROC22 was initially downregulated, and subsequently upregulated with an extended duration of *S. scitamineum* challenge.

## Discussion

### Identification and annotation of miRNAs and high-throughput sequencing of sugarcane sRNAs

YA05-179 is a BC4 generation of sugarcane and *Eranthus arundinacus* with high smut-resistance, whereas ROC22 is a highly smut-susceptible variety. These two sugarcane varieties have completely opposite disease-resistance performance and thus are good models for gene regulation during sugarcane smut infection. A previous study has shown that under natural stress, the disease symptoms of sugarcane smut usually occur two to four months after the dissemination of pathogens [62], with the phenocritical period significantly later than the gene regulation period. To maintain a consistent physiological and biochemical condition, the present study used the artificial inoculation with 5 × 10^6^ spores/mL smut spore suspension [63]. Su et al. [8] showed that the logarithmic growth period of the pathogens was at 48 h after the artificial inoculation of sugarcane smut pathogens, and the stationary phase was at 96 h post-inoculation, suggesting that the critical period for physiological and biochemical reactions in sugarcane in response to the smut disease was the first 48 h after inoculation.

Solexa sequencing technology, HiSeq sequencing system, 454 sequencing, and sequencing by oligonucleotide ligation and detection (SOLiD) sequencing technology [64], which are relatively mature and optimized and have been widely used in the analysis of plant miRNA expression profiles [65], the screening of differentially expressed miRNAs under biotic and abiotic stresses [66], the identification of miRNAs associated with growth and development [67], and the discovery of novel miRNAs with new functions [68]. In the present study, we used HiSeq sequencing system to identify differentially expressed miRNAs in sugarcane after challenging with the pathogen for smut disease. The number of clean reads obtained from each RCK, RT, YACK, and YAT contained <1% of contaminated sequences and therefore, 99% of the sequences were clean reads. Statistical data of analyses of the sequence length and the common and specific sequences showed that the sequencing quality of each sample was relatively high, with good overall consistency among different samples.

The genetic background of sugarcane is complicated, and genome analysis of sugarcane is currently in its preliminary stages [69]. ESTs are only a part of the annotation results in the database, suggesting that most of the unknown sequences have not been characterized. Sequences detected from the four sugarcane samples were not directly matched with the miRNA sequences in the miRBase database. For the prediction of novel miRNAs, we combined our sequencing data with the Sugarcane_Unigene sequences of the sugarcane transcriptome database, which was provided by our laboratory. The sequencing results of the present study were comparable to the entries in the sugarcane transcriptome database [47] and the suga﻿rcane EST in GenBank, thereby indicating that our findings were reliable. Statistical analysis of base distribution of novel miRNAs demonstrated that the most common base of novel miRNAs of 21 and 22 nt in length was U. In addition, nucleotide 11 of candidate miRNA sequences of the four samples was A. A previous study revealed that plant miRNAs mostly act via the degradation of target mRNAs [70]. The cleavage of this degradation occurred very precisely at nucleotide 10 or 11 of the matched miRNAs [70]. In addition, miRNAs bound to the argonaut (AGO) protein later complementarily bound to the mRNA 3′ untranslated region of the target gene, thereby cleaving or degrading the target gene [71]. In the present study, the 5′ end of 21- and 22-nt novel miRNA sequences preferentially harbored the U base, indicating that these novel miRNA sequences might play an important role in regulating gene expression after smut pathogen challenge. In addition, nucleotide 11 of candidate miRNA sequences in the four samples tended to be A, which was consistent with the bias of cleavage sites of degradation, as well as the statistical results of the base, thereby suggesting that the prediction method used in the present study was feasible. The above results showed that the quality of the measured data was high, thus meeting the requirements for subsequent analysis.

### Screening and analysis of differentially expressed miRNAs by using qRT-PCR

High-throughput sequencing generates massive data on miRNAs. Therefore, the present study exclusively focused on screening of differentially expressed miRNAs after *S. scitamineum* challenge. The present study identified individual differences in miRNA expression in the two sugarcane varieties, YA05-179 and ROC22, after the challenge of sugarcane smut p﻿athogen, as well as differences between the two varieties. In terms of individual differences of each variety after *S. scitamineum* challenge, ROC22 had 231 differentially expressed miRNAs, which included 35 significantly expressed miRNAs (|log_2_-ratio| >1, *P* <0.01), and the read count of miR2199, miR2916, miR5077, miR5813, miR6300, and miR894 was >10,000. Among the 208 differentially expressed miRNAs screened in YA05-179, 11 miRNAs showed significant individual differences in miRNA expression, of which the read count of miR894 was also >10,000. In terms of differences between two sugarcane varieties, when ROC22 and YA05-179 were subjected to smut disease, the differentially expressed miRNAs screened from the individual known miRNAs were mostly the same. However, miR397-3p (−1.54-fold) was upregulated in YA05-179 only, although not very high. Eight known miRNAs, including miR1077-3p, miR262, miR5212-3p, miR5020a, miR1144a.1, miR5657, miR6149-3p, and miR536, were specifically expressed in the smut resistant-genotype, YA05-179. Cluster analysis of expression patterns of known miRNAs in the two sugarcane varieties showed that most miRNAs changed in the same way in the two varieties following treatment (either upregulated in both or downregulated in both), with only differences found in the degree of the changes. Some regulatory modes of the miRNAs were inconsistent, although not significant. The present study identified 48 novel miRNAs that were differentially expressed in ROC22, of which 16 were upregulated. Forty-one differentially expressed novel miRNAs were detected in YA05-179, of which six were upregulated. The differentially expressed miRNAs with significant expression levels in both control and treated groups were almost specifically expressed in a single sugarcane variety, with differentially expressed novel miRNAs determined to be the majority. No novel miRNA was differentially expressed in both sugarcane varieties. In addition, to ensure the reliability of expression profiling data, miRNAs obtained from high-throughput sequencing should be verified in plant tissues, commonly by qRT-PCR [72]. The present study validated the 27 expression profiles correspond to 16 differentially expressed miRNAs in ROC22 and YA05-179 (Fig. 5). Except for the unmatched expression level and sequencing results of miR5261 and miR397-3p in YA05-179 and miR5077 and novel_mir_99 in ROC22, the expression patterns (up- or downregulated) of the remaining miRNAs and their corresponding sequencing results were consistent, suggesting that the sequencing results of the present study were reliable. Whereas the minor discrepancy between the qRT-PCR expression levels and sequencing results may possibly be due to different software and algorithms used in processing the vast amount of data generated from high-throughput sequencing [73, 74]. Overall, the results indicated that the high-throughput sequencing was a powerful tool for discovering novel and differentially expressed miRNAs in sugarcane after *S. scitamineum* challenge, and it is also anticipated that more replicates in further work should help to ensure the accuracy of the sequencing results.

Previous studies have demonstrated that the regulatory networks of the target genes of plant miRNAs in response to the environmental stress are centrally located, thereby affecting a variety of regulatory signals [67, 75]. In the present study, we focused on the possible regulatory roles of differentially expressed known and novel miRNAs in sugarcane after smut pathogen challenge. Seven miRNAs, including miR894, miR394a, miR7545, miR397-3p, miR5261, miR5783, and miR948, were co-expressed in both sugarcane varieties after the inoculation of *S. scitamineum* and showed different expression patterns. This study also identified a specifically expressed miRNA, namely, miR5261, in YA05-179 and ROC22, with highly significant fold changes in the expression and a relatively wide range of differential fluctuations in expression levels. Five miRNAs, including miR408-3p, novel_mir_32, novel_mir_133, novel_mir_99, and novel_mir_58 were only detected in a single sugarcane variety after *S. scitamineum* inoculation. However, the specific roles of differentially expressed miRNAs on the target genes in resisting *S. scitamineum* after sugarcane smut infection remain unclear and need to be further investigated.

### Prediction and expression analysis of target genes of differentially expressed miRNAs

Sugarcane is a highly heterozygous allopolyploid and aneuploid crop [69]. The present study performed a comprehensive analysis of existing transcriptome sequences and our experimental sequencing results. However, because whole-genome sequencing of sugarcane has not been completed [69], majority of the sRNA sequences during miRNA annotation remained unknown. The results of the present study indicated that although the number of predicted target genes of the whole miRNAs in the YA05-179 and ROC22 varieties was relatively equivalent, that of differentially expressed known miRNAs in YA05-179 (127) was less than that observed in ROC22 (814). On the other hand, the predicted target gene number of differentially expressed novel miRNAs in YA05-179 (1754) was significantly higher than that in ROC22 (457). The number of differentially expressed known miRNAs in YA05-179 (10) after *S. scitamineum* challenge was slightly lower than that in ROC22 (32). Predictive analysis of differentially expressed novel miRNA candidates in both sugarcane varieties demonstrated that only a few novel miRNA candidates were co-expressed in both sugarcane varieties. On the other hand, most of the novel miRNA candidates were specifically expressed in a single variety and majority of these were upregulated. Prediction results of the above target genes indicated that novel miRNAs screened from YA05-179 were more significant in the evaluation of the molecular mechanism underlying the interaction between sugarcane and *S. scitamineum*.

Functional analysis of target genes of miRNAs is the most direct approach in studying the function of miRNAs [20]. Bioinformatics analysis can effectively predict the target genes of miRNAs, including its functions [76]. In the present study, we focused on identifying the regulatory role of differentially expressed miRNAs, with functional analysis and annotation of potential target genes from the GO and KEGG pathways. In the three GO categories, namely, biological processes, cellular components, and molecular functions, the classifications of predicted target genes of the differentially expressed novel miRNAs in both sugarcane varieties were similar to the sequencing data on the predicted target genes of differentially expressed known miRNAs. The target gene distribution in the biological processes was mainly associated with cellular processes and metabolic processes. KEGG pathway enrichment analysis demonstrated that the predicted target genes of differentially expressed miRNAs participated in a series of biochemical pathways or disease resistance-related physiological, metabolic, and signal transduction pathways such as plant-pathogen interaction, peroxisome, apoptosis, phagosome, cutin, suberine and wax biosynthesis, plant hormone signal transduction, MAPK signaling pathway, calcium signaling pathway, zeatin biosynthesis, and brassinosteroid biosynthesis. Although the predicted target genes of the identified differentially expressed miRNAs from different sugarcane varieties were not the same, its response processes almost covered every aspect throughout the life course, and its metabolic regulatory pathways were identical. Previous studies have also shown that the molecular mechanism of the interaction between sugarcane and *S. scitamineum* was regulated by multigenic network systems, and the pathogen of sugarcane smut also activated a variety of smut-resistance metabolic pathways [77, 78]. In addition, our findings prompted us to speculate that miRNAs post-transcriptionally regulate mRNAs, which are consistent with the results of previous studies [77, 78]. Upregulation of miRNAs may result in the degradation of target genes or the downregulation of miRNAs may promote the overexpression of target genes, thereby changing a number of metabolic or signal transduction pathways.

### Expression analysis of several predicted target genes using qRT-PCR

Prediction and functional analysis of the target genes of differentially expressed miRNAs are efficient approaches in studying the functions of miRNAs [20]. In the present study, we used qRT-PCR to evaluate the correlation between miRNAs and its predicted target genes and analyzed the expression patterns of the target genes of differentially expressed miRNAs in the same sugarcane variety at different time points after *S. scitamineum* inoculation. In total, 15 predicted target genes of 12 miRNAs were detected, including 11 predicted target genes of eight known miRNAs and four predicted target genes of four novel miRNAs (Fig. 6). We combined the miRNA expression patterns and the target gene expression patterns to analyze the quantitative expression of target genes at four different time points and its corresponding miRNA expression.

qRT-PCR analysis (Fig. 6) showed that except for the inconsistent expression between the predicted target gene, CF569707, and its corresponding miRNA, miR5783, ten out of 11 predicted target genes of known miRNAs had basically identical negatively regulated mode after 12 h and reached the highest degree of matching at 48 h. In particular, the expression patterns of three corresponding target genes of miR948 were identical, suggesting that the expression of these genes was highly consistent with that of the miRNAs. These results also indicated that the regulatory effect of the corresponding genes of miRNAs after *S. scitamineum* challenge was maximized at 48 h after post-inoculation. However, this is a negative regulatory effect. The above results also showed that the expression enrichment prediction of known miRNAs and its predicted target genes in the present study was reliable. Our prediction of biochemical, metabolic, or signal transduction pathways associated with the miRNAs and its predicted target genes might provide novel insights on future anti-smut research studies. In addition, the negatively regulated role in quantitative expression between the novel miRNAs and its predicted target genes was not extremely high. The expression of CF570081 and CF57094 was anti-correlated with miRNA results at 48 and 96 h but the expression of CF575522 and CF576305 was not. It is possible that these potential target genes are regulated by more than one miRNAs at the translational level [79]. Meanwhile, due to the lacking of relevant research and also the lack of sugarcane genomic information, many of the novel miRNAs identified in the present study are being reported for the first time. These miRNAs do represent a portion of novel miRNAs involved with smut pathogen challenge, however it needs more samples or sequencing coverage to extract more reliable and functional novel miRNAs. Further improvement in methods and strategies may be necessary for the prediction and functional analysis of novel miRNAs, such as mismatches of nucleotide sequences of miRNA, which may cause errors in gene targeting [60].

### Analyses of predicted target gene functions and smut resistance-related metabolic pathways

**Fig. 7:**
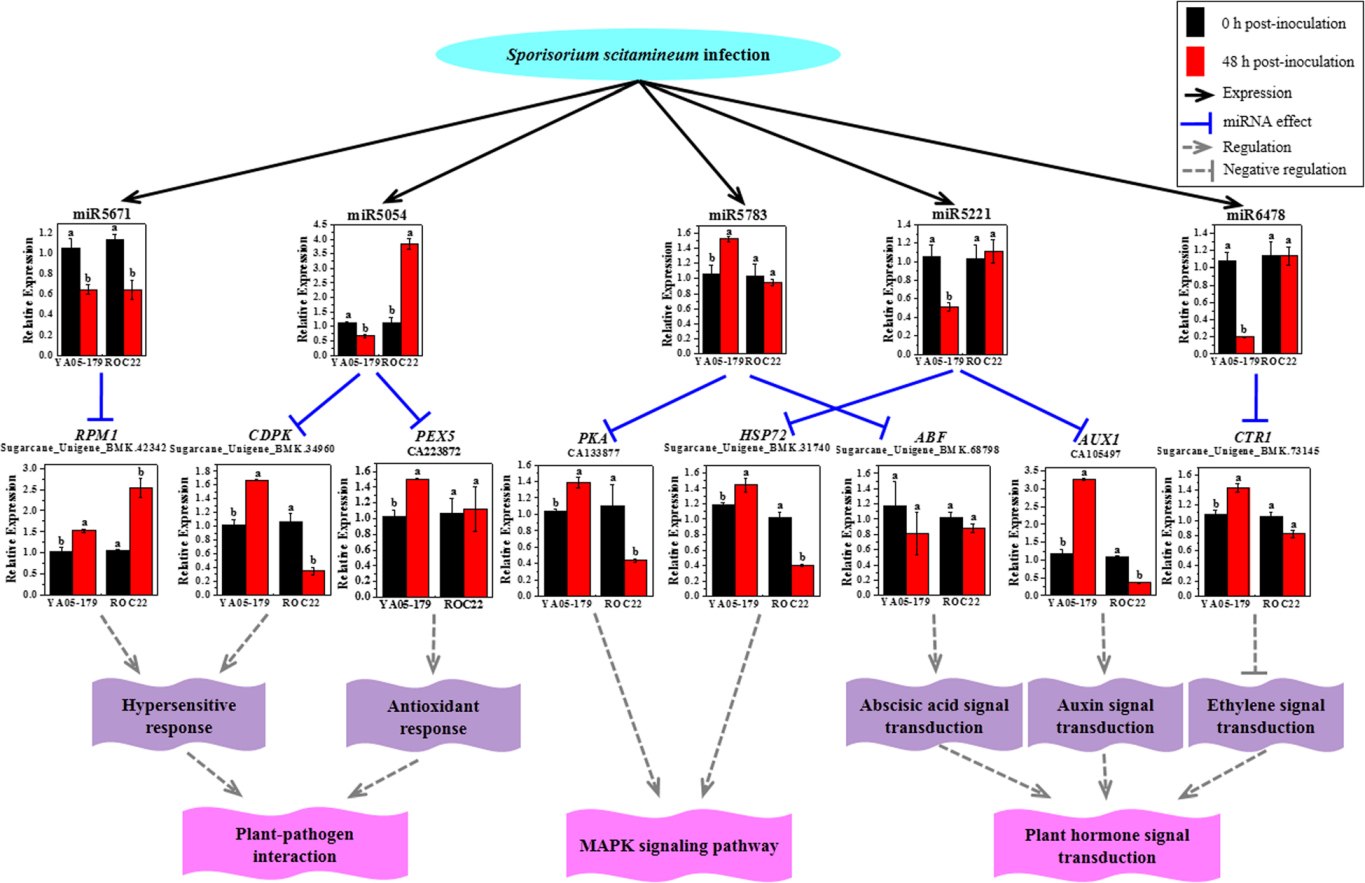
A proposed regulatory network of partial miRNAs in sugarcane after *Sporisorium scitamineum* challenge. The gene expression profiles of five differentially expressed miRNAs, including miR5671, miR5054, miR5783, miR5221, and miR6478, as well as eight of their target genes, including *RPM1* (Sugarcane_Unigene_BMK.42342), *CDPK* (Sugarcane_Unigene_BMK.34960), *PEX5* (CA223872), *PKA* (CA133877), *HSP72* (Sugarcane_Unigene_BMK.31740), *ABF* (Sugarcane_Unigene_BMK.68798), *AUX1* (CA105497), and *CTR1* (Sugarcane_Unigene_BMK.73145), were validated by qRT-PCR. The sugarcane buds from ROC22 and YA05-179 inoculated with distilled water and *S. scitamineum* at 0 and 48 h were used for qRT-PCR samples. The expression profiles of the miRNAs were normalized to the *5S rRNA* expression level and represented as means of three replicates (*n* = 3) ± standard error. The expression profiles of the target genes were normalized to the glyceraldehyde-3-phosphate dehydrogenase (*GAPDH*) expression level and represented as means of three replicates (*n* = 3) ± standard error. Different lowercase letters indicate a significant difference, as determined by Duncan’s new multiple range test (*P*-value <0.05). RPM1, effector-triggered immune receptor; CDPK, calmodulin-independent protein kinase; PEX5, peroxisome 5; PKA, protein kinase A; HSP72, heat shock protein 72; ABF, ABA-responsive element-binding protein; AUX1, auxin transporter; CTR1, constitutive triple-response 1

A previous study demonstrated that in response to pathogen infection, the appearance, physiological, and biochemical changes in host cultivars are ultimately caused by the disruptions at the molecular level [80]. Disease-susceptible varieties show relatively slow and weak responses and signals to infection, whereas the responses of signals of disease-resistant varieties are relatively rapid and strong [80]. For these reasons, the disease-resistant plant varieties could combat most of the damages inflicted by the pathogen, as well as prevent its proliferation and further spread [80]. In the present study, the predicted target genes of differentially expressed miRNAs in ROC22 and YA05-179 were determined to participate in several metabolic pathways after *S. scitamineum* challenge. Notably, three possible pathways associated with smut pathogen stress, including plant-pathogen interaction pathway, MAPK signalling pathway, and plant hormone signal transduction, were chosen for further analysis. The expression profiles of five crucial miRNAs and eight of their predicted target genes in YA05-179 and ROC22 after *S. scitamineum* challenge for 48 h were confirmed by qRT-PCR (Fig. 7).

#### MAPK signaling pathway in plants

When plant cells are exposed to environmental stress and receive hormonal signals, MAPK cascade pathway was activated to induce the expression of specific transcription factors in the nucleus and functional gene that would, in turn, activate other protein kinases in the cytoplasm to ultimately trigger the plant cell to elicit physiological and biochemical responses [81]. A previous study has shown that when rice crops were infected by *Pyricularia grisea*, several genes belonging to the MAPK pathway were upregulated [82]. In the present study, the predicted target genes of some miRNAs participated in the MAPK cascade pathway. As reported, expression of the ﻿prot﻿ein kinase A (*PKA*)﻿ gene significantly inhibits the activities of several members of the MAPK family, thereby blocking the MAPK signaling pathway [83]. Wheareas, the target *PKA* gene (CA133877) of miR5783 was significantly upregulated (1.39-) in the smut-resistant genotype YA05-179, but downregulated (0.44-) in the smut-susceptible genotype ROC22 (Fig. 7). Similarly, miR5783 was also significantly upregulated (1.52-) in YA05-179 and downregulated (0.95-) in ROC22 but not at significant level. We speculated that miR5783 might positively regulate *PKA* expression, thereby regulating related genes in the MAPK pathway. Heat shock protein 72 (HSP72) is a chaperone protein that plays an important regulatory role in the cells of various organisms [84]. During adverse conditions such as high temperature, endotoxin, and oxidative stress, organisms stimulate and induce the synthesis of HSP72 [84]. In the present study, miR5221 was significantly downregulated (0.51-) in YA05-179 and slightly upregulated (1.11-) in ROC22 which was not significant. Expression level of the predicted target gene of miR5221, *HSP72* (Sugarcane_Unigene_BMK.31740), was significantly increased (1.44-) in YA05-179 and significantly decreased (0.40-) in ROC22, suggesting that miR5221 may play a negative regulation role in *HSP72* expression to combat smut infestation.

#### The signal transduction pathways of plant hormones

Plant hormones play an important role in the response to environmental stresses [85]. The most common plant hormones include auxin (namely indole-3-acetic acid, IAA), cytokinin (CK), abscisic acid (ABA), gibberellin acid (GA), ethylene (ET), brassinosteroid (BR), salicylic acid (SA), jasmonic acid (JA), and polyamines. A previous study has shown that plant hormones play a critical role in plant defenses against pathogens [86]. During growth and development, plants generate regulatory defense responses to abiotic stresses, which include the endogenous hormones, SA, ET, and JA. During abiotic stress in plants, ABA interacts with the SA, JA, and ET-regulated signal transduction pathways and negatively regulates the resistance of abiotic stress in plants [87]. IAA induces the transient expression of some genes in response to abiotic stress [88]. *AUX1* (auxin transporter) is an auxin influx carrier protein that plays a role in cellular transport in the plant root system [89]. Overexpression of *AUX1* accelerates the transportation of IAA, which regulates the expression of a stress-related gene, C-repeat binding factor (CBF) [90]. In the present study, the predicted target genes of the differentially expressed miRNAs participate in a variety of hormone signaling pathways, thereby suggesting that these miRNA-regulated target genes play critical roles in signal transduction pathways involving plant hormones. Among these miRNAs, miR5221 targeted the *AUX1* gene and negatively regulate its expression in both sugarcane genotypes. In Fig. 7, *AUX1* (CA105497) was significantly upregulated (3.27-) in YA05-179 and downregulated (0.37-) in ROC22. Constitutive triple-response 1 (*CTR1*) is a gene involved in the ET signal transduction pathway. A previous study showed that the amino terminus of CTR1 could combine with the ET receptor to form a complex, which negatively regulates the ET response [91]. Our analysis indicated that the expression of miR6478 was obviously repressed (0.20-) in YA05-179 but remained stable (1.14-) in ROC22. The *CTR1* gene (Sugarcane_Unigene_BMK.73145), one of the predicted target genes of miR6478, was significantly upregulated (1.43-) in YA05-179 and only slightly downregulated (0.82-) in ROC22. This result indicated that miR6478 might negatively regulate *CTR1* expression and affected the ET signal transduction pathway. ABA pathway has been reported to be a negative regulator of plant disease resistance [92]. *ABF* is a binding factor of ABA-responsive element-binding protein (*AREB*) [93]. *AREB/ABF* transcription factors are ABA-responsive element-binding proteins that regulating the expression of ABA-related genes [93]. In the present study, miR5783 targeted the *ABF* gene (Sugarcane_Unigene_BMK.68798) and slightly downregulated (0.81- and 0.88-) it in both YA05-179 and ROC22 however the level was not significant. This finding suggested that miR5783 might play a negative regulation role in *ABF* in YA05-179, but a positive regulation role in *ABF* in ROC22. Based on the above findings, we speculated that the upregulated and downregulated expression of differentially expressed miRNAs reflect the close relationship of the auxin signal transduction pathway, ABA signal transduction pathway, and ET signal transduction pathway in sugarcane during *S. scitamineum* infection.

#### Pathway of plant-pathogen interaction

In plant-pathogen interaction, plants have a variety of defense mechanisms against different pathogens [94], including hypersensitive responses (HR), changes in enzymatic activities, and the accumulation of defense proteins [95]. A previous study has indicated that catalase plays an important role in plant defense response, stress response, and in regulating redox balance in cells [96]. In the present study, miR5054 was downregulated (0.64-) in YA05-179 and significantly upregulated (3.83-) in ROC22. The expression of one miR5054 targeted gene, peroxisome 5 (*PEX5*, CA223872), was significantly increased (1.51-) in YA05-179 and nearly remained stable (1.12-) in ROC22. Calcium-dependent and calmodulin-independent protein kinase (*CDPK*) is a key protein gene that involved in the mechanism of disease resistance in plants [97]. Overexpression of *CDPK* triggers the primary reaction of active cell necrosis. In addition, hydrogen peroxide also activates CDPK and enhances the expression of the *CDPK* gene [98]. In the present study, *CDPK* gene (Sugarcane_Unigene_BMK.34960), one of the predicted target genes of miR5054, was significantly upregulated (1.67-) in YA05-179 and downregulated (0.34-) in ROC22, revealing an opposite expression trend compared to that of miR5054 in both sugarcane genotypes. These results revealed that there might be a negatively regulated mode between miR5054 and *PEX5 or CDPK* gene. The formation of HR depends on the interaction between plant disease resistance gene products and the avirulence (*Avr*) gene product of the corresponding pathogen [99]. *RPM1* (effector-triggered immune receptor) is a type of R-gene-mediated plant disease resistance, whose overexpression induces HR in plants [99]. In the present study, miR5671 was determined to target *RPM1*. We found that the expression of *RPM1* (Sugarcane_Unigene_BMK.42342) was significantly increased (1.53- and 2.54-) in YA05-179 and ROC22 after *S. scitamineum* challenge. Meanwhile, miR5671 was significantly downregulated (0.65- and 0.65) in both sugarcane genotypes, suggesting the negative regulation of *RPM1* in sugarcane smut resistance.

## Conclusions

This is the first study that has employed high-throughput sequencing technology to identify and establish the expression profiles of various sugarcane miRNAs that are associated with *S. scitamineum* challenge. The post-transcriptional miRNA regulatory mechanism in the compatible and incompatible interactions between sugarcane and *S. scitamineum* was then systemically evaluated, which enriched and deepened our knowledge in the molecular mechanism underlying sugarcane resistance to smut disease. The present study has presented various regulatory pathways that are affected by smut infection, as well as generated a regulatory network of some miRNAs in sugarcane post *S. scitamineum* infection (Fig. 7).

## Additional files

Additional file 1: Table S1. The forward primers of qRT-PCR performed to validate the 20 selected differentially expressed miRNAs. (DOCX 20 kb)

Additional file 2: Table S2. The primers of qRT-PCR performed to validate 23 selected miRNA target genes. (DOC 41 kb)

Additional file 3: Table S3. The filtering results of high-through sequencing data in the four libraries. (DOC 40 kb)

Additional file 4:Figure S1. Length distribution of the unique sRNA sequences in the four libraries. RCK and YACK: ROC22 and YA05-179 under sterile water stress after 48 h, respectively; RT and YAT: ROC22 and YA05-179 under *Sporisorium scitamineum* stress after 48 h, respectively. (TIF 35 kb)

Additional file 5: Table S4. The statistics of types and total number of repetitive sequence of the sRNAs in the four libraries. (DOC 69 kb)

Additional file 6: Table S5. The matching results of sRNAs among non-coding RNAs in the four libraries by Genbank search. (DOC 30 kb)

Additional file 7: Table S6. The matching results of sRNAs among non-coding RNAs in the four libraries by Rfam search. (DOC 31 kb)

Additional file 8: Figure S2. The distribution of first nucleotide bias (A) and the nucleotide bias at each position (B) of the novel miRNAs in the four libraries. (A) Each color in the figure showed the miRNA tags whose first base was a certain base. Height of bar was proportional to the frequency of the corresponding base at the given length from 20 to 23 nt. (B) Each color in the figure showed the miRNA tags whose certain base was a certain base. Height of bar was proportional to the frequency of the corresponding base at the given position from 1 to 23 nt. RCK and YACK: ROC22 and YA05-179 under sterile water stress after 48 h, respectively; RT and YAT: ROC22 and YA05-179 under *Sporisorium scitamineum* stress after 48 h, respectively. (ZIP 279 kb)

Additional file 9: Table S7. The significantly differentially expressed known miRNAs in the RT/RCK. (DOC 70 kb)

Additional file 10: Table S8. The significantly differentially expressed known miRNAs in the YAT/YACK. (DOC 37 kb)

Additional file 11:Table S9. The significantly differentially expressed novel miRNAs in the RT/RCK. (DOC 42 kb)

Additional file 12: Table S10. The significantly differentially expressed novel miRNAs in the YAT/YACK. (DOC 32 kb)

Additional file 13: Table S11. Prediction of target genes of known and novel miRNAs. (DOC 30 kb)

Additional file 14: Table S12. The prediction results for partial target genes of differentially expressed miRNAs. (DOC 41 kb)

Additional file 15: Figure S3. GO categories and distribution of known miRNAs targets in RT/RCK (A) and YAT/YACK (B), respectively. RCK and YACK: ROC22 and YA05-179 under sterile water stress after 48 h, respectively; RT and YAT: ROC22 and YA05-179 under *Sporisorium scitamineum* stress after 48 h, respectively. (TIF 3774 kb)

Additional file 16: Figure S4. GO categories and distribution of novel miRNAs targets in RT/RCK (A) and YAT/YACK (B), respectively. RCK and YACK: ROC22 and YA05-179 under sterile water stress after 48 h, respectively; RT and YAT: ROC22 and YA05-179 under *Sporisorium scitamineum* stress after 48 h, respectively. (TIF 1606 kb)

Additional file 17: Table S13. KEGG analysis of predicted target genes of known miRNAs in RT/RCK. (DOC 58 kb)

Additional file 18: Table S14. KEGG analysis of predicted target genes of known miRNAs in YAT/YACK. (DOC 34 kb)

Additional file 19: Table S15. KEGG analysis of predicted target genes of novel miRNAs in RT/RCK. (DOC 57 kb)

Additional file 20: Table S16. KEGG analysis of predicted target genes of novel miRNAs in YAT/YACK. (DOC 58 kb)

## Abbreviations

2DE: Two-dimensional gel electrophoresis
ABA: Abscisic acid
AGO: Argonaut
ANOVA: Analysis of Variance
APS1: ATP sulfurylase 1
AREB: ABA-responsive element-binding protein
AUX1: Auxin transporter
Avr: Avirulence
BLASTn: Basic local alignment search tool
BR: Brassinosteroid
CBF: C-repeat binding factor
cDNA-AFLP: cDNA-amplified fragment length polymorphism
CDPK: Calmodulin-independent protein kinase
CK: Cytokinin
CTR1: Constitutive triple-response 1
ESTs: Expressed sequence tags
ET: Ethylene
GA: Gibberellin acid
GAPDH: Glyceraldehyde-3-phosphate dehydrogenase
GO: Gene Ontology
Hc-Pro: Helper component-proteinase
HR: Hypersensitive responses
HSP72: Heat shock protein 72
IAA: Indole-3-acetic acid
iTRAQ: Isobaric tags for relative and absolute quantitation
KEGG: Kyoto Encyclopedia of Genes and Genomes
LAMP: Loop-mediated isothermal amplification
MALDI-TOF MS: Matrix-assisted laser desorption/ionization time-of-flight mass spectrometry
MAPK: Mitogen-activated protein kinase
miRNA: microRNA
PEX5: Peroxisome 5
piRNA: Piwi-interacting RNA
PKA: Kinase A
qRT-PCR: Quantitative real-time PCR
RPM: Reads per million
RPM1: Effector-triggered immune receptor
rRNA: Ribosomal RNA
SA: Salicylic acid
scRNA: Small cytoplasmic RNA
siRNAs: Small interfering RNAs
snoRNA: Small nucleolar RNA
snRNA: Small nuclear RNA
SOLiD: Sequencing by oligonucleotide ligation and detection
SRA: Sequence read archive
sRNA: Non-coding small RNA
srpRNA: Signal recognition particle RNA
SSH: Suppression subtractive hybridization
tRNA: Transfer RNA
