# Supplementary material for: Small RNA sequencing reveals a role for sugarcane miRNAs and their targets in response to Sporisorium scitamineum infection
Source: BMC Genomics. 2017 Apr 24;18:325. doi: 10.1186/s12864-017-3716-4 (PMC5404671; doi:10.1186/s12864-017-3716-4)
Supplement: Supplementary file 1 — The forward primers of qRT-PCR performed to validate the 20 selected differentially expressed miRNAs. (DOCX 20 kb) [file 12864_2017_3716_MOESM1_ESM.docx]

**Small RNA sequencing reveals a role for sugarcane miRNAs and their targets in response to *Sporisorium scitamineum* infection**

**Yachun Su**

**E-mail:** **[syc2009mail@163.com](mailto:syc2009mail@163.com)**

**Yuye Zhang**

**E-mail: guang_mi@163.com**

**Ning Huang**

**E-mail: hning2012@126.com**

**Feng Liu**

**E-mail: 18359162091@163.com**

**Weihua Su**

**E-mail: suweihua2016@126.com**

**Liping Xu**

**E-mail:** [**xlpmail@126.com**](mailto:xlpmail@126.com)

**Waqar Ahmad**

**E-mail: wahmadhu@gmail.com**

**Qibin Wu**

**E-mail: wqbaidqq@163.com**

**Jinlong Guo**

**E-mail: jl.guo@163.com**

**Youxiong Que***

**E-mail:** [**queyouxiong@126.com**](mailto:queyouxiong@126.com)

Key Laboratory of Sugarcane Biology and Genetic Breeding, Ministry of Agriculture, Fujian

Agriculture and Forestry University, Fuzhou 350002, China

***Correspondence should be addressed to** [queyouxiong@126.com](mailto:queyouxiong@126.com)

**The full postal address of the submitting author Youxiong Que is as follows:** Key Laboratory of

Sugarcane Biology and Genetic Breeding, Ministry of Agriculture, Fujian Agriculture and Forestry

University, Fuzhou 350002, China

**Table S1.** The forward primers of qRT-PCR performed to validate the 20 selected differentially expressed miRNAs

| **miRNA name** | **Primer sequences (5’~3’)** |
| --- | --- |
| miR394a | TTGGCATTCTGTCCACCTCC |
| miR5066 | AGGTGTATAAGTGGGAGCCC |
| miR5059 | TTCCTGGGCAGCAACAC |
| miR5261 | CTTGTGGAAGGCTTTGGCTA |
| miR7545 | CGGGCTTGAAGAAATTAGAGTGCT |
| miR894 | GTTTCACGTCGGGTTCACCA |
| miR948 | TGTGGTCGTGGGTTCGGGAA |
| miR5783 | ATTTAAGACGAGGACGAGGAGGACGCC |
| miR5077 | TTCACGTCGGGTTCACCAAA |
| miR6300 | CGGGCGTCGTTGTAGTATAGTGGT |
| miR408-3p | CTGCACTGCCTCTTCCCTGGC |
| miR397-3p | TCACCGGCGCTGCACTCAATT |
| miR5671 | CATGGTGGTGACGGGTGAC |
| miR5054 | GTTCCCCACGGACGGCGCCA |
| miR5221 | AACGAGATGGTGTTTTACTT |
| miR6478 | CCGACCTTAGCTCAGTTGGTA |
| novel_mir_133 | GGGTTTTAGGGTTTAGGGTTT |
| novel_mir_58 | GCCCGTTTGAATAAGACGAGTGGTCA |
| novel_mir_99 | CAAGCAAGTTGGGATAGGCTAGAA |
| novel_mir_32 | TCGTCGCCGTCGTCGTCGTC |
| 5S rRNA | CGATCCCATTCCGACCTCGATA |
