# Supplementary material for: Small RNA sequencing reveals a role for sugarcane miRNAs and their targets in response to Sporisorium scitamineum infection
Source: BMC Genomics. 2017 Apr 24;18:325. doi: 10.1186/s12864-017-3716-4 (PMC5404671; doi:10.1186/s12864-017-3716-4)
Supplement: Supplementary file 2 — The primers of qRT-PCR performed to validate 23 selected miRNA target genes. (DOC 41 kb) [file 12864_2017_3716_MOESM2_ESM.doc]

**Table S2. The primers of qRT-PCR performed to validate 23 selected miRNA target genes**

| **Target gene name** | **miRNA name** | **Forward primer sequences (5’-3’)** | **Reverse primer sequences (5’-3’)** |
| --- | --- | --- | --- |
| CF574674 | miR394a | GGAAGGAGGTGGACAGAATG | GTAACAAGGCGGTTCATACG |
| Sugarcane_Unigene_BMK.105 | miR397-3p | GCCACCGCCTGATGAGAC | CTTGCTCCACCCCATCCTAC |
| CF573748 | miR408-3p | CACGAGTCTCAAGTACAC | AACTGCTCATCTGCTTAG |
| CF577228 | miR5261 | ACTTCATGCTCTCCTCATACG | CGCTGCTGGTGATCTCTG |
| AA961317 | miR5783 | TTCTTACTCGTCTTGGATGG | GCTATGTTCAGGAGGAAGG |
| CF569707 | miR5783 | CGGTTCGTCCTCGTCTTC | TTGACTGATGATAAACAACTTGAC |
| GT757759 | miR7545 | CTTTCGCAGTTGTTCGTCTTTC | GCCATCGCTCAGGATACATTAG |
| CF573424 | miR894 | CTTGGAGATGATGGACGAGTC | TGACGCTCAGATGTGAATGG |
| CF569809 | miR948 | GTAGTAGGAGGAGGAGAG | AAGGAACCATCAGAGAAC |
| CF573595 | miR948 | TGGACTCATTCAACAGGAATACAG | CATATAATCTCAGTGGCTGCTCTC |
| CF577206 | miR948 | ATTGCCTGCCTTCTCTAAG | GTCATCTAGCCACCAACTC |
| CF570940 | novel_mir_133 | CGCCAGAGTAGAGAGATG | AATCACCTTGACGGAGAC |
| CF570081 | novel_mir_32 | AGGACGAGGAGGAGAGGAG | GCCGCCAATGAAGACCTG |
| CF576305 | novel_mir_58 | ACGCTCTCTCAAATACTGCTGTC | CCGCCACAAGACCACTGAAC |
| CF575522 | novel_mir_99 | GGATAGGAGCACTTGGAGATTGG | CAACAACAACAGCAACAACATAGC |
| Sugarcane_Unigene_BMK.42342 | miR5671 | TCACCACCAGGCTCCTCAACC | TCCTCGCCCACTGCTCCACA |
| Sugarcane_Unigene_BMK.34960 | miR5054 | ATATTATTTCCCTTCTCCATCCATGC | CGTCGGGTTCGGGCTGAT |
| CA223872 | miR5054 | GAGCATCCGACCAAACCATT | AAGATACAGGGACATCAGTCTCATTC |
| CA133877 | miR5783 | AGTGCAGCATGTCTCAGTTCATC | CCACGCTATCTCCCAAATGTC |
| Sugarcane_Unigene_BMK.68798 | miR5783 | GTGGTTTGACAAGCGAGAATAGTGAT | GTTGCTGATGCTGGGAGTGGA |
| Sugarcane_Unigene_BMK.31740 | miR5221 | TGTCATCACCGTGCCTGTCTACTTC | ATGTCCATCCTCCCGTGTTTCC |
| CA105497 | miR5221 | TGGAAACCGCAGAAGTTCAAGC | GCCAGGAACCAGATAGGCACAA |
| Sugarcane_Unigene_BMK.73145 | miR6478 | ACAAGCGACCCGTCTTTCAC | CCCAGTTAGCCGACTTAGGA |
| *GAPDH* | / | CACGGCCACTGGAAGCA | TCCTCAGGGTTCCTGATGCC |
