# Supplementary material for: Small RNA sequencing reveals a role for sugarcane miRNAs and their targets in response to Sporisorium scitamineum infection
Source: BMC Genomics. 2017 Apr 24;18:325. doi: 10.1186/s12864-017-3716-4 (PMC5404671; doi:10.1186/s12864-017-3716-4)
Supplement: Supplementary file 3 — The filtering results of high-through sequencing data in the four libraries. (DOC 40 kb) [file 12864_2017_3716_MOESM3_ESM.doc]

**Table S3.** The filtering results of high-through sequencing data in the four libraries

| **Category** | **RCK** | |  | **RT** | |  | **YACK** | |  | **YAT** | |
| --- | --- | --- | --- | --- | --- | --- | --- | --- | --- | --- | --- |
|  | **Number** | **Percentage** |  | **Number** | **Percentage** |  | **Number** | **Percentage** |  | **Number** | **Percentage** |
| total reads | 36,857,130 | / |  | 28,081,198 | / |  | 27,749,621 | / |  | 28,632,700 | / |
| high quality | 36,681,670 | 100% |  | 27,963,445 | 100% |  | 27,631,201 | 100% |  | 28,513,596 | 100% |
| 3' adapter null | 22,157 | 0.06% |  | 14,269 | 0.05% |  | 12,878 | 0.05% |  | 15,838 | 0.06% |
| insert null | 7,863 | 0.02% |  | 4,358 | 0.02% |  | 3,751 | 0.01% |  | 4,511 | 0.02% |
| 5' adapter contaminants | 72,315 | 0.20% |  | 81,810 | 0.29% |  | 47,064 | 0.17% |  | 51,731 | 0.18% |
| smaller than 18nt | 180,370 | 0.49% |  | 47,510 | 0.17% |  | 101,795 | 0.37% |  | 149,552 | 0.52% |
| polyA | 2,377 | 0.01% |  | 2,526 | 0.01% |  | 1,245 | 0.00% |  | 1,733 | 0.01% |
| clean reads | 36,396,588 | 99.22% |  | 27,812,972 | 99.46% |  | 27,464,468 | 99.40% |  | 28,290,231 | 99.22% |

RCK and YACK: ROC22 and YA05-179 under sterile water stress after 48 h, respectively; RT and YAT: ROC22 and YA05-179 under *Sporisorium scitamineum* stress after 48 h, respectively.
