# Supplementary figures and images for: Small RNA sequencing reveals a role for sugarcane miRNAs and their targets in response to Sporisorium scitamineum infection
Source: BMC Genomics. 2017 Apr 24;18:325. doi: 10.1186/s12864-017-3716-4 (PMC5404671; doi:10.1186/s12864-017-3716-4)

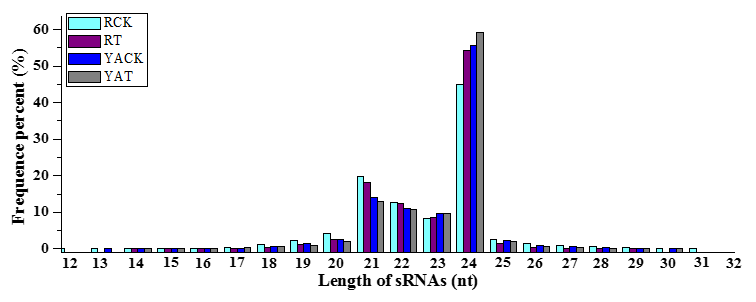

Supplement: Supplementary file 4 — Length distribution of the unique sRNA sequences in the four libraries. RCK and YACK: ROC22 and YA05-179 under sterile water stress after 48 h, respectively; RT and YAT: ROC22 and YA05-179 under Sporisorium scitamineum stress after 48 h, respectively. (TIF 35 kb) [file 12864_2017_3716_MOESM4_ESM.tif]

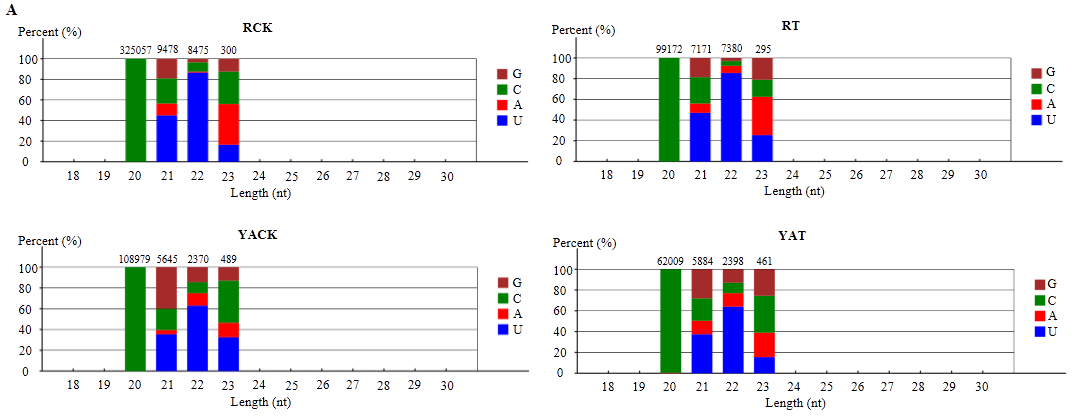

Supplement: Supplementary file 8 — The distribution of first nucleotide bias (A) and the nucleotide bias at each position (B) of the novel miRNAs in the four libraries. (A) Each color in the figure showed the miRNA tags whose first base was a certain base. Height of bar was proportional to the frequency of the corresponding base at the given length from 20 to 23 nt. (B) Each color in the figure showed the miRNA tags whose certain base was a certain base. Height of bar was proportional to the frequency of the corresponding base at the given position from 1 to 23 nt. RCK and YACK: ROC22 and YA05-179 under sterile water stress after 48 h, respectively; RT and YAT: ROC22 and YA05-179 under Sporisorium scitamineum stress after 48 h, respectively. (ZIP 279 kb) [file 12864_2017_3716_MOESM8_ESM.zip › Figure S2/Figure S2A.tif]

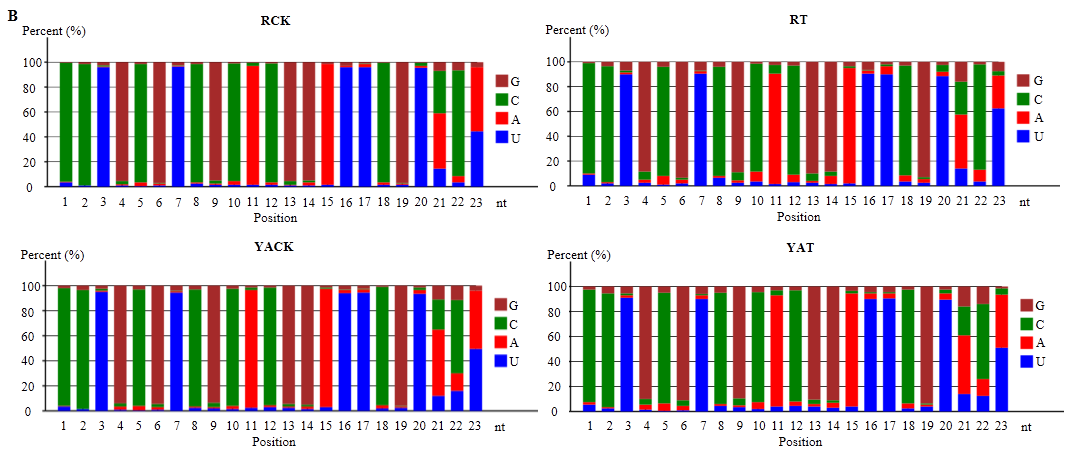

Supplement: Supplementary file 8 — The distribution of first nucleotide bias (A) and the nucleotide bias at each position (B) of the novel miRNAs in the four libraries. (A) Each color in the figure showed the miRNA tags whose first base was a certain base. Height of bar was proportional to the frequency of the corresponding base at the given length from 20 to 23 nt. (B) Each color in the figure showed the miRNA tags whose certain base was a certain base. Height of bar was proportional to the frequency of the corresponding base at the given position from 1 to 23 nt. RCK and YACK: ROC22 and YA05-179 under sterile water stress after 48 h, respectively; RT and YAT: ROC22 and YA05-179 under Sporisorium scitamineum stress after 48 h, respectively. (ZIP 279 kb) [file 12864_2017_3716_MOESM8_ESM.zip › Figure S2/Figure S2B.tif]

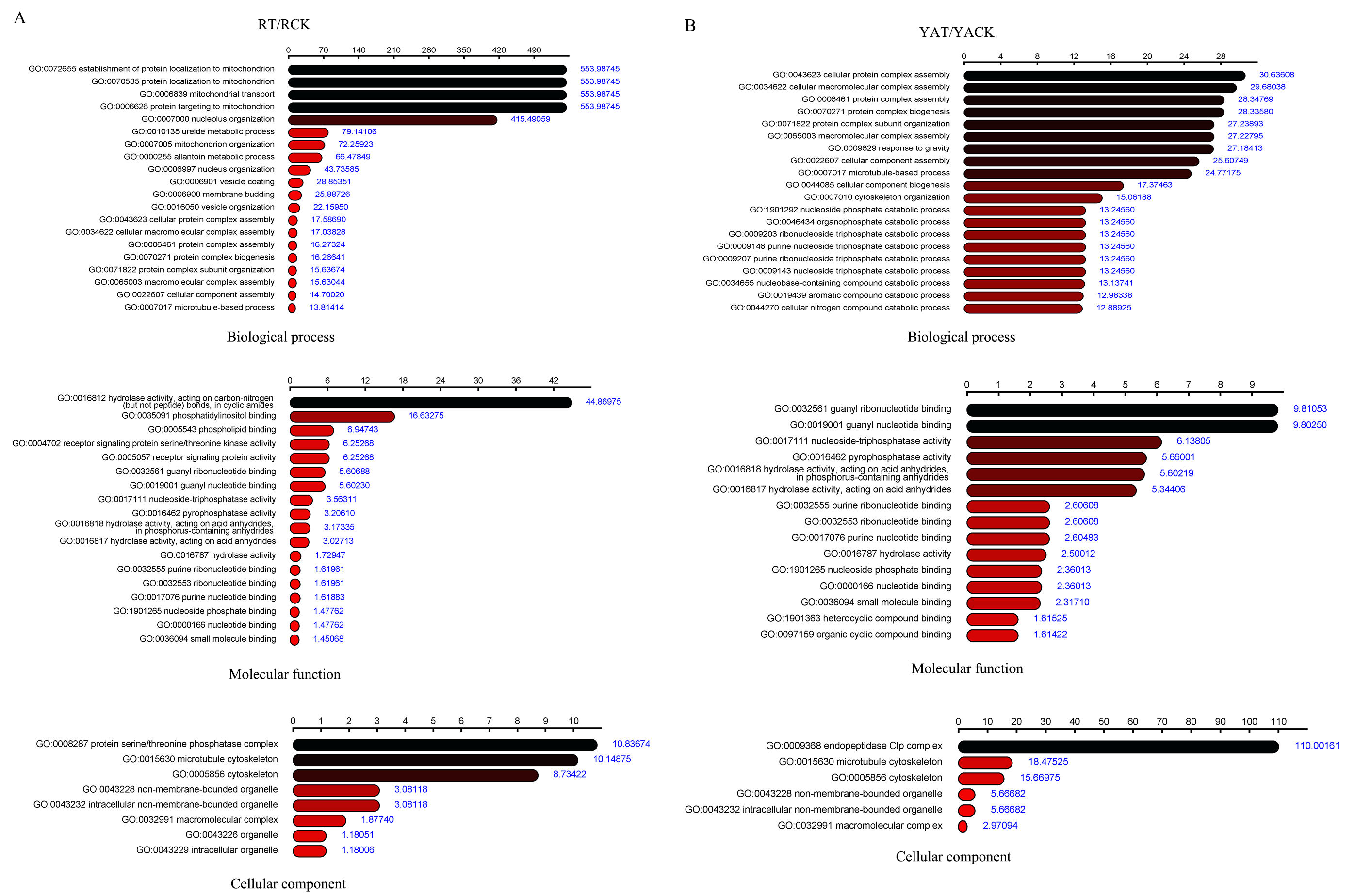

Supplement: Supplementary file 15 — GO categories and distribution of known miRNAs targets in RT/RCK (A) and YAT/YACK (B), respectively. RCK and YACK: ROC22 and YA05-179 under sterile water stress after 48 h, respectively; RT and YAT: ROC22 and YA05-179 under Sporisorium scitamineum stress after 48 h, respectively. (TIF 3774 kb) [file 12864_2017_3716_MOESM15_ESM.tif]

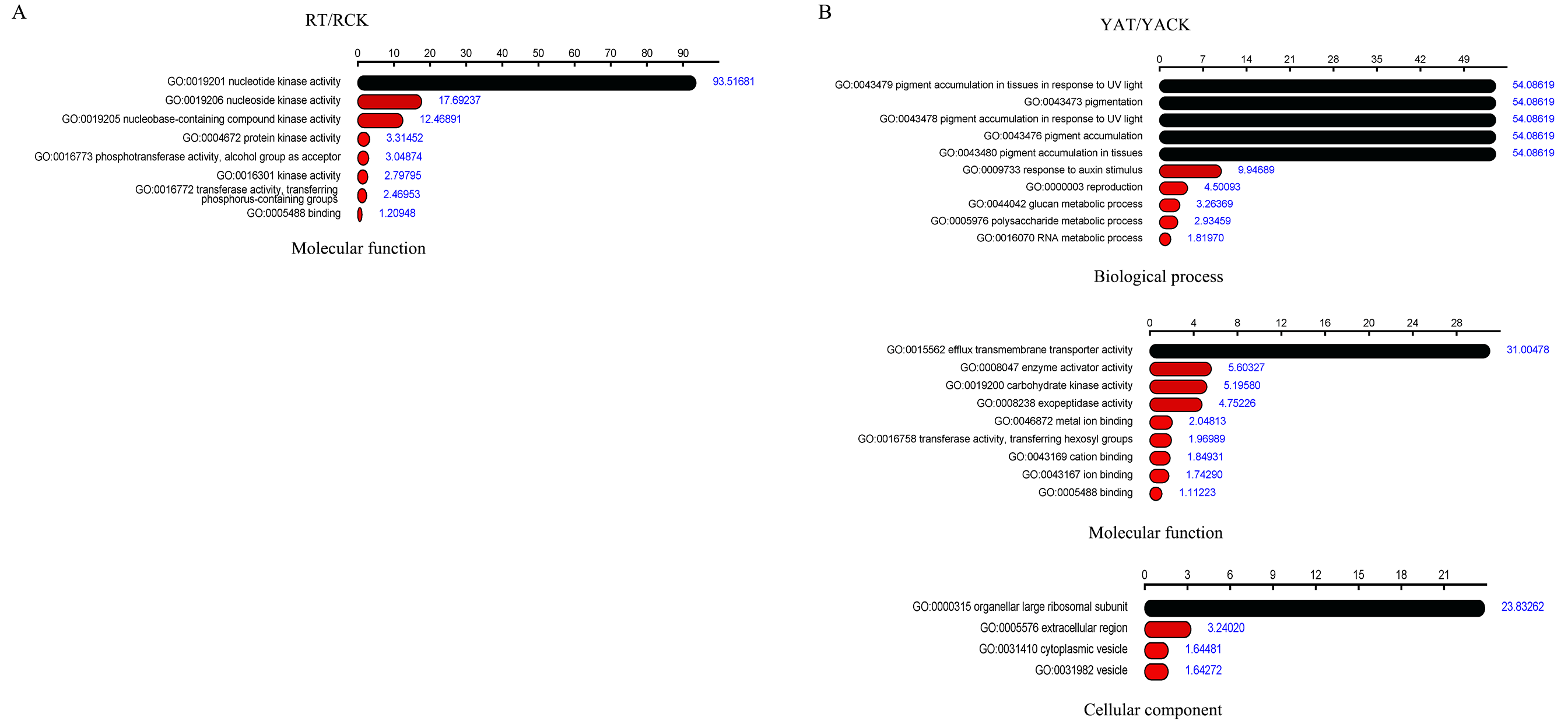

Supplement: Supplementary file 16 — GO categories and distribution of novel miRNAs targets in RT/RCK (A) and YAT/YACK (B), respectively. RCK and YACK: ROC22 and YA05-179 under sterile water stress after 48 h, respectively; RT and YAT: ROC22 and YA05-179 under Sporisorium scitamineum stress after 48 h, respectively. (TIF 1606 kb) [file 12864_2017_3716_MOESM16_ESM.tif]
