# Supplementary material for: Small RNA sequencing reveals a role for sugarcane miRNAs and their targets in response to Sporisorium scitamineum infection
Source: BMC Genomics. 2017 Apr 24;18:325. doi: 10.1186/s12864-017-3716-4 (PMC5404671; doi:10.1186/s12864-017-3716-4)
Supplement: Supplementary file 5 — The statistics of types and total number of repetitive sequence of the sRNAs in the four libraries. (DOC 69 kb) [file 12864_2017_3716_MOESM5_ESM.doc]

**Table S4.** The statistics of types and total number of repetitive sequence of the sRNAs in the four libraries

| **Type** | **RCK** | | **RT** | | **YACK** | | **YAT** | |
| --- | --- | --- | --- | --- | --- | --- | --- | --- |
|  | **Unique sRNAs** | **Total sRNAs** | **Unique sRNAs** | **Total sRNAs** | **Unique sRNAs** | **Total sRNAs** | **Unique sRNAs** | **Total sRNAs** |
| DNA/TcMar-Mariner:1 | 1 | 1 | 0 | 0 | 0 | 0 | 0 | 0 |
| DNA/hAT-Charlie:1 | 1 | 1 | 1 | 1 | 0 | 0 | 0 | 0 |
| DNA/hAT-Tip100:0 | 78 | 112 | 73 | 103 | 40 | 77 | 46 | 80 |
| DNA/hAT-Tip100:1 | 185 | 588 | 172 | 544 | 139 | 517 | 157 | 585 |
| LINE/CR1:0 | 0 | 0 | 2 | 3 | 0 | 0 | 0 | 0 |
| LINE/CR1:1 | 3 | 3 | 2 | 2 | 0 | 0 | 1 | 1 |
| LINE/L1:0 | 20 | 22 | 10 | 12 | 18 | 18 | 8 | 9 |
| LINE/L1:1 | 10 | 10 | 15 | 17 | 11 | 12 | 12 | 16 |
| LINE/L2:0 | 4 | 4 | 1 | 1 | 5 | 5 | 7 | 7 |
| LINE/L2:1 | 1 | 1 | 0 | 0 | 1 | 1 | 0 | 0 |
| LTR/ERV1:0 | 23 | 47 | 16 | 30 | 25 | 47 | 20 | 36 |
| LTR/ERV1:1 | 15 | 21 | 17 | 26 | 9 | 9 | 11 | 12 |
| LTR/ERVK:0 | 0 | 0 | 1 | 1 | 0 | 0 | 0 | 0 |
| LTR/ERVL-MaLR:1 | 1 | 1 | 0 | 0 | 0 | 0 | 0 | 0 |
| LTR/ERVL:0 | 2 | 2 | 1 | 1 | 1 | 1 | 0 | 0 |
| LTR/ERVL:1 | 1 | 1 | 0 | 0 | 0 | 0 | 0 | 0 |
| LTR/Gypsy:0 | 3 | 3 | 2 | 2 | 2 | 2 | 3 | 3 |
| LTR/Gypsy:1 | 0 | 0 | 0 | 0 | 1 | 1 | 2 | 2 |
| RC/Helitron:0 | 66 | 125 | 65 | 127 | 59 | 108 | 71 | 133 |
| RC/Helitron:1 | 24 | 36 | 31 | 36 | 29 | 40 | 26 | 26 |
| SINE/Deu:0 | 72 | 300 | 68 | 347 | 79 | 278 | 71 | 285 |
| SINE/Deu:1 | 154 | 9,834 | 131 | 3,209 | 146 | 4,318 | 132 | 2,415 |
| Satellite:0 | 0 | 0 | 1 | 1 | 1 | 1 | 0 | 0 |
| Satellite:1 | 4 | 7 | 1 | 4 | 2 | 3 | 3 | 3 |
| Unknown:0 | 7 | 9 | 4 | 5 | 3 | 3 | 5 | 6 |
| Unknown:1 | 24 | 72 | 20 | 54 | 26 | 90 | 25 | 85 |
| rRNA:0 | 6,658 | 102,946 | 6,301 | 117,252 | 5,643 | 41,962 | 5,856 | 43,603 |
| rRNA:1 | 57,275 | 3,360,267 | 35,578 | 896,714 | 46,817 | 1,376,950 | 38,361 | 758,935 |
| snRNA:0 | 30 | 42 | 29 | 35 | 38 | 49 | 42 | 59 |
| snRNA:1 | 439 | 3,422 | 230 | 1,605 | 247 | 1019 | 229 | 1,239 |
| srpRNA:1 | 2 | 2 | 0 | 0 | 0 | 0 | 0 | 0 |
| tRNA:0 | 61 | 83 | 44 | 59 | 75 | 108 | 62 | 92 |
| tRNA:1 | 1,829 | 77,705 | 1,011 | 22,897 | 1,345 | 29,254 | 1,069 | 18,026 |

RCK and YACK: ROC22 and YA05-179 under sterile water stress after 48 h, respectively; RT and YAT: ROC22 and YA05-179 under *Sporisorium scitamineum* stress after 48 h, respectively.
