# Supplementary material for: Small RNA sequencing reveals a role for sugarcane miRNAs and their targets in response to Sporisorium scitamineum infection
Source: BMC Genomics. 2017 Apr 24;18:325. doi: 10.1186/s12864-017-3716-4 (PMC5404671; doi:10.1186/s12864-017-3716-4)
Supplement: Supplementary file 6 — The matching results of sRNAs among non-coding RNAs in the four libraries by Genbank search. (DOC 30 kb) [file 12864_2017_3716_MOESM6_ESM.doc]

**Table S5. The matching results of sRNAs among non-coding RNAs in the four libraries by Genbank search**

| **Category** | **RCK sRNAs** | **RT sRNAs** | **YACK sRNAs** | | **YAT sRNAs** |
| --- | --- | --- | --- | --- | --- |
| rRNA | 1,439,391 | 417,991 | 659,761 | 390,107 | |
| tRNA | 65,872 | 28,700 | 32,686 | 33,628 | |
| other | 34,891,325 | 27,366,281 | 26,772,021 | 27,866,496 | |
| total | 36,396,588 | 27,812,972 | 27,464,468 | 28,290,231 | |

rRNA, ribosomal RNA; tRNA, transfer RNA. RCK and YACK: ROC22 and YA05-179 under sterile water stress after 48 h, respectively; RT and YAT: ROC22 and YA05-179 under *Sporisorium scitamineum* stress after 48 h, respectively.
