# Supplementary material for: Small RNA sequencing reveals a role for sugarcane miRNAs and their targets in response to Sporisorium scitamineum infection
Source: BMC Genomics. 2017 Apr 24;18:325. doi: 10.1186/s12864-017-3716-4 (PMC5404671; doi:10.1186/s12864-017-3716-4)
Supplement: Supplementary file 7 — The matching results of sRNAs among non-coding RNAs in the four libraries by Rfam search. (DOC 31 kb) [file 12864_2017_3716_MOESM7_ESM.doc]

**Table S6.** The matching results of sRNAs among non-coding RNAs in the four libraries by Rfam search

| **Category** | **RCK sRNAs** | **RT sRNAs** | **YACK sRNAs** | **YAT sRNAs** |
| --- | --- | --- | --- | --- |
| rRNA | 2,054,081 | 679,605 | 841,027 | 504,347 |
| snRNA | 25,283 | 11,608 | 12,854 | 12,340 |
| snoRNA | 13,334 | 5,793 | 6,200 | 5,375 |
| tRNA | 1,538,638 | 459,977 | 572,497 | 310,253 |
| other | 32,765,252 | 26,655,989 | 26,031,890 | 27,457,916 |
| total | 36,396,588 | 27,812,972 | 27,464,468 | 28,290,231 |

RCK and YACK: ROC22 and YA05-179 under sterile water stress after 48 h, respectively; RT and YAT: ROC22 and YA05-179 under *Sporisorium scitamineum* stress after 48 h, respectively.
