# Supplementary material for: Small RNA sequencing reveals a role for sugarcane miRNAs and their targets in response to Sporisorium scitamineum infection
Source: BMC Genomics. 2017 Apr 24;18:325. doi: 10.1186/s12864-017-3716-4 (PMC5404671; doi:10.1186/s12864-017-3716-4)
Supplement: Supplementary file 9 — The significantly differentially expressed known miRNAs in the RT/RCK. (DOC 70 kb) [file 12864_2017_3716_MOESM9_ESM.doc]

**Table S7. The significantly differentially expressed known miRNAs in the RT/RCK**

| **miRNA name** | **RCK**  **read count** | **RT**  **read count** | **RCK**  **read normalize** | **RT**  **read normalize** | **fold-change**  **(log_2_ RT/RCK)** | **p-value** | **sig-lable** |
| --- | --- | --- | --- | --- | --- | --- | --- |
| miR1158 | 1,030 | 33 | 28.30 | 1.19 | -4.58 | 7.583E-204 | ** |
| miR1310 | 1,639 | 338 | 45.03 | 12.15 | -1.89 | 3.126E-136 | ** |
| miR158a | 65 | 23 | 1.79 | 0.83 | -1.11 | 0.0010 | ** |
| miR1861d | 395 | 13 | 10.85 | 0.47 | -4.54 | 1.100E-78 | ** |
| miR2199 | 14,901 | 3,489 | 409.41 | 125.45 | -1.71 | 0 | ** |
| miR2916 | 12,397 | 3,982 | 340.61 | 143.17 | -1.25 | 0 | ** |
| miR390a | 625 | 1,179 | 17.17 | 42.39 | 1.30 | 4.718E-79 | ** |
| miR390a-3p | 23 | 64 | 0.63 | 2.30 | 1.86 | 1.212E-08 | ** |
| miR394a | 23 | 64 | 0.63 | 2.30 | 1.86 | 1.212E-08 | ** |
| miR399b-5p | 24 | 49 | 0.66 | 1.76 | 1.42 | 0.431E-05 | ** |
| miR408-3p | 50 | 19 | 1.37 | 0.68 | -1.01 | 0.0077 | ** |
| miR5054 | 11,446 | 2,219 | 314.48 | 79.78 | -1.98 | 0 | ** |
| miR5056 | 21 | 37 | 0.58 | 1.33 | 1.21 | 0.0018 | ** |
| miR5059 | 7,073 | 2,145 | 194.33 | 77.12 | -1.33 | 0 | ** |
| miR5066 | 593 | 116 | 16.29 | 4.17 | -1.97 | 5.366E-53 | ** |
| miR5072 | 954 | 174 | 26.21 | 6.26 | -2.07 | 1.001E-89 | ** |
| miR5077 | 22,444 | 6,028 | 616.65 | 216.73 | -1.51 | 0 | ** |
| miR5152-3p | 100 | 30 | 2.75 | 1.08 | -1.35 | 1.716E-06 | ** |
| miR5221 | 373 | 13 | 10.25 | 0.47 | -4.45 | 1.411E-73 | ** |
| miR5242 | 416 | 13 | 11.43 | 0.47 | -4.61 | 1.412E-83 | ** |
| miR5261 | 117 | 32 | 3.21 | 1.15 | -1.48 | 2.703E-08 | ** |
| miR5368 | 180 | 65 | 4.95 | 2.34 | -1.08 | 5.998E-08 | ** |
| miR5498 | 45 | 78 | 1.24 | 2.80 | 1.18 | 7.704E-06 | ** |
| miR5641 | 24 | 40 | 0.66 | 1.44 | 1.13 | 0.0021 | ** |
| miR5671 | 6,207 | 2,128 | 170.54 | 76.51 | -1.16 | 2.333E-249 | ** |
| miR5783 | 1,350 | 2,743 | 37.09 | 98.62 | 1.41 | 1.043E-204 | ** |
| miR5813 | 15,737 | 4,356 | 432.38 | 156.62 | -1.47 | 0 | ** |
| miR6182 | 134 | 210 | 3.68 | 7.55 | 1.04 | 4.124E-11 | ** |
| miR6300 | 22,128 | 6,611 | 607.97 | 237.70 | -1.35 | 0 | ** |
| miR6478 | 2,338 | 714 | 64.24 | 25.67 | -1.32 | 6.828E-117 | ** |
| miR6480 | 112 | 8 | 3.08 | 0.29 | -3.42 | 3.394E-19 | ** |
| miR7545 | 1,358 | 443 | 37.31 | 15.93 | -1.23 | 2.172E-61 | ** |
| miR8155 | 895 | 299 | 24.59 | 10.75 | -1.19 | 2.477E-39 | ** |
| miR894 | 71,303 | 18,612 | 1,959.06 | 669.18 | -1.55 | 0 | ** |
| miR948 | 405 | 107 | 11.13 | 3.85 | -1.53 | 1.700E-26 | ** |

**: fold-change (log_2_-ratio) >1 or fold-change (log_2_-ratio) <-1, and p-value <0.01. RCK and RT: ROC22 under sterile water and *Sporisorium scitamineum* stress after 48 h, respectively.
