# Supplementary material for: Small RNA sequencing reveals a role for sugarcane miRNAs and their targets in response to Sporisorium scitamineum infection
Source: BMC Genomics. 2017 Apr 24;18:325. doi: 10.1186/s12864-017-3716-4 (PMC5404671; doi:10.1186/s12864-017-3716-4)
Supplement: Supplementary file 10 — The significantly differentially expressed known miRNAs in the YAT/YACK. (DOC 37 kb) [file 12864_2017_3716_MOESM10_ESM.doc]

**Table S8.** The significantly differentially expressed known miRNAs in the YAT/YACK

| **miRNA name** | **YACK**  **read count** | **YAT**  **read count** | **YACK**  **read normalize** | **YAT**  **read normalize** | **fold-change**  **(log_2_** **YAT/YACK)** | **p-value** | **sig-lable** |
| --- | --- | --- | --- | --- | --- | --- | --- |
| miR1310 | 567 | 290 | 20.64 | 10.25 | -1.01 | 2.035E-23 | ** |
| miR390a-3p | 19 | 41 | 0.69 | 1.45 | 1.07 | 0.0063 | ** |
| miR394a | 96 | 263 | 3.50 | 9.30 | 1.41 | 3.457E-18 | ** |
| miR397-3p | 31 | 11 | 1.13 | 0.39 | -1.54 | 0.0014 | ** |
| miR5059 | 2,603 | 1,288 | 94.78 | 45.53 | -1.06 | 3.237E-109 | ** |
| miR5066 | 264 | 117 | 9.61 | 4.14 | -1.22 | 2.707E-15 | ** |
| miR5152-3p | 56 | 13 | 2.04 | 0.46 | -2.15 | 5.280E-08 | ** |
| miR5261 | 53 | 20 | 1.93 | 0.71 | -1.45 | 5.677E-05 | ** |
| miR6478 | 942 | 454 | 34.30 | 16.05 | -1.09 | 5.640E-43 | ** |
| miR7545 | 532 | 255 | 19.37 | 9.01 | -1.10 | 2.910E-25 | ** |
| miR894 | 33,261 | 15,877 | 1,211.06 | 561.22 | -1.11 | 0 | ** |

**: fold-change (log_2_-ratio) >1 or fold-change (log_2_-ratio) <-1, and p-value <0.01. YACK and YAT: YA05-179 under sterile water and *Sporisorium scitamineum* stress after 48 h, respectively.
