# Supplementary material for: Small RNA sequencing reveals a role for sugarcane miRNAs and their targets in response to Sporisorium scitamineum infection
Source: BMC Genomics. 2017 Apr 24;18:325. doi: 10.1186/s12864-017-3716-4 (PMC5404671; doi:10.1186/s12864-017-3716-4)
Supplement: Supplementary file 11 — The significantly differentially expressed novel miRNAs in the RT/RCK. (DOC 42 kb) [file 12864_2017_3716_MOESM11_ESM.doc]

**Table S9.** The significantly differentially expressed novel miRNAs in the RT/RCK

| **miRNA name** | **RCK**  **read count** | **RT**  **read count** | **RCK**  **read normalize** | **RT**  **read normalize** | **fold-change**  **(log_2_ RT/RCK)** | **p-value** | **sig-lable** |
| --- | --- | --- | --- | --- | --- | --- | --- |
| novel_mir_11 | 1,029 | 0 | 28.27 | 0.01 | -11.46 | 2.322E-254 | ** |
| novel_mir_110 | 0 | 32 | 0.01 | 1.15 | 6.85 | 2.044E-12 | ** |
| novel_mir_112 | 0 | 446 | 0.01 | 16.04 | 10.65 | 7.631E-163 | ** |
| novel_mir_113 | 0 | 37 | 0.01 | 1.33 | 7.06 | 3.116E-14 | ** |
| novel_mir_116 | 0 | 40 | 0.01 | 1.44 | 7.17 | 2.533E-15 | ** |
| novel_mir_118 | 0 | 228 | 0.01 | 8.20 | 9.68 | 1.240E-83 | ** |
| novel_mir_120 | 0 | 48 | 0.01 | 1.73 | 7.438 | 3.139E-18 | ** |
| novel_mir_133 | 0 | 34 | 0.01 | 1.22 | 6.93 | 3.834E-13 | ** |
| novel_mir_23 | 39 | 0 | 1.07 | 0.01 | -6.74 | 2.751E-10 | ** |
| novel_mir_4 | 97 | 13 | 2.67 | 0.47 | -2.51 | 9.431E-13 | ** |
| novel_mir_58 | 336 | 0 | 9.23 | 0.01 | -9.85 | 1.650E-83 | ** |
| novel_mir_67 | 126 | 211 | 3.46 | 7.59 | 1.13 | 1.147E-12 | ** |
| novel_mir_69 | 52 | 19 | 1.43 | 0.68 | -1.06 | 0.0045 | ** |
| novel_mir_80 | 65 | 0 | 1.79 | 0.01 | -7.48 | 1.070E-16 | ** |
| novel_mir_89 | 162,526 | 49,586 | 4,465.42 | 1,782.84 | -1.32 | 0 | ** |
| novel_mir_9 | 298 | 0 | 8.19 | 0.01 | -9.68 | 3.854E-74 | ** |
| novel_mir_99 | 0 | 34 | 0.01 | 1.22 | 6.93 | 3.834E-13 | ** |

**: fold-change (log_2_-ratio) >1 or fold-change (log_2_-ratio) <-1, and p-value <0.01. If the original miRNA expression in a library was zero, the normalized read count of this miRNA was adjusted to 0.01 in the library for further calculation [48–50]. RCK and YACK: ROC22 and YA05-179 under sterile water stress after 48 h, respectively; RT and YAT: ROC22 and YA05-179 under *Sporisorium scitamineum* stress after 48 h, respectively.
