# Supplementary material for: Small RNA sequencing reveals a role for sugarcane miRNAs and their targets in response to Sporisorium scitamineum infection
Source: BMC Genomics. 2017 Apr 24;18:325. doi: 10.1186/s12864-017-3716-4 (PMC5404671; doi:10.1186/s12864-017-3716-4)
Supplement: Supplementary file 12 — The significantly differentially expressed novel miRNAs in the YAT/YACK. (DOC 32 kb) [file 12864_2017_3716_MOESM12_ESM.doc]

**Table S10.** The significantly differentially expressed novel miRNAs in the YAT/YACK

| **miRNA name** | **YACK**  **read count** | **YAT**  **read count** | **YACK**  **read normalize** | **YAT**  **read normalize** | **fold-change**  **(log_2_ YAT/YACK)** | **p-value** | **sig-lable** |
| --- | --- | --- | --- | --- | --- | --- | --- |
| novel_mir_134 | 136 | 0 | 4.95 | 0.01 | -8.95 | 1.486E-42 | ** |
| novel_mir_170 | 0 | 34 | 0.01 | 1.20 | 6.91 | 9.738E-11 | ** |
| novel_mir_184 | 0 | 45 | 0.01 | 1.59 | 7.31 | 5.589E-14 | ** |
| novel_mir_187 | 0 | 228 | 0.01 | 8.06 | 9.65 | 6.719E-68 | ** |
| novel_mir_192 | 0 | 56 | 0.01 | 1.98 | 7.63 | 3.208E-17 | ** |
| novel_mir_32 | 33 | 0 | 1.20 | 0.01 | -6.91 | 7.009E-11 | ** |

**: fold-change (log_2_-ratio) >1 or fold-change (log_2_-ratio) <-1, and p-value <0.01. If the original miRNA expression in a library was zero, the normalized read count of this miRNA was adjusted to 0.01 in the library for further calculation [48–50]. RCK and YACK: ROC22 and YA05-179 under sterile water stress after 48 h, respectively; RT and YAT: ROC22 and YA05-179 under *Sporisorium scitamineum* stress after 48 h, respectively.
