# Supplementary material for: Small RNA sequencing reveals a role for sugarcane miRNAs and their targets in response to Sporisorium scitamineum infection
Source: BMC Genomics. 2017 Apr 24;18:325. doi: 10.1186/s12864-017-3716-4 (PMC5404671; doi:10.1186/s12864-017-3716-4)
Supplement: Supplementary file 13 — Prediction of target genes of known and novel miRNAs. (DOC 30 kb) [file 12864_2017_3716_MOESM13_ESM.doc]

**Table S11. Prediction of target genes of known and novel miRNAs**

| **Sample name** | **known miRNAs** | | | **novel miRNAs** | | |
| --- | --- | --- | --- | --- | --- | --- |
|  | **miRNA** number | **Target gene number** | **Number of target gene loci** | **miRNA** number | **Target gene number** | **Number of target gene loci** |
| RCK | 209 | 4,588 | 5,348 | 128 | 9,899 | 33,682 |
| RT | 209 | 4,588 | 5,348 | 133 | 11,357 | 33,212 |
| RT/RCK | 32 | 814 | 839 | 15 | 457 | 613 |
| YACK | 207 | 4,586 | 5,346 | 101 | 7,321 | 15,512 |
| YAT | 208 | 4,587 | 5,347 | 111 | 12,642 | 32,521 |
| YAT/YACK | 10 | 127 | 127 | 6 | 1,754 | 1,811 |

RCK and YACK: ROC22 and YA05-179 under sterile water stress after 48 h, respectively; RT and YAT: ROC22 and YA05-179 under *Sporisorium scitamineum* stress after 48 h, respectively. With reference to the Sugarcane_Unigenetranscriptome database (65,852 unigenes) established by our previous studytranscriptome analysis in ROC22 and YA05-179 post-*S. scitamineum* infection for 24 h, 48 h and 120 h [47] and the sugarcane EST in GenBank, the present study used the psRNATarget online software to predict the target genes of the known miRNAs and the novel miRNAs.
