# Supplementary material for: Small RNA sequencing reveals a role for sugarcane miRNAs and their targets in response to Sporisorium scitamineum infection
Source: BMC Genomics. 2017 Apr 24;18:325. doi: 10.1186/s12864-017-3716-4 (PMC5404671; doi:10.1186/s12864-017-3716-4)
Supplement: Supplementary file 14 — The prediction results for partial target genes of differentially expressed miRNAs. (DOC 41 kb) [file 12864_2017_3716_MOESM14_ESM.doc]

**Table S12. The prediction results for partial target genes of differentially expressed miRNAs**

| **miRNA name** | **Target gene ID** | **Annotation of the target genes** |
| --- | --- | --- |
| miR394a | CF574996 | MCSA142A02 Maturing Sugarcane Stem Lambda ZIPLOX Library, unknown function |
|  | CF574674 | MCSA118C02 Maturing Sugarcane Stem Lambda ZIPLOX Library (MCS), unknown function |
| miR5077 | Sugarcane_Unigene_BMK.61960  Sugarcane_Unigene_BMK.62421 | hypothetical protein  hypothetical protein |
| miR408-3p | CF573748 | similar to amine oxidase |
|  | CF569691 | similar to plantacyanin |
| miR397-3p | Sugarcane_Unigene_BMK.105 | hypothetical protein |
| miR5261 | CF577228 | similar to tubulin |
|  | CA111920 | similar to CDPK (Calcium-dependent and calmodulin-independent protein kinase) |
| miR5783 | CF569707 | similar to glycine-rich RNA binding protein |
|  | AA961317 | similar to tubulin beta-1 chain |
|  | CA133877 | similar to PKA (protein kinaes A) |
| miR7545 | GT757759 | similar to 18S ribosomal RNA gene |
| miR894 | CF573424 | MCSA070B02 Maturing Sugarcane Stem Lambda ZIPLOX Library, unknown function |
| miR948 | CF577206 | similar to protein kinase |
|  | CF573595 | similar to annexin |
|  | CF571852 | similar to protein kinase |
|  | CF569809 | similar to small GTP-binding protein |
| novel_mir_133 | CF570940 | similar to glycine-rich RNA binding protein |
| novel_mir_58 | CF576305 | similar to receptor-like protein kinase |
| novel_mir_80 | CF575522 | similar to zinc finger protein |
|  | CF574703 | similar to zinc finger protein |
|  | CF571944 | similar to zinc finger protein |
| novel_mir_99 | HS075930 | similar to Hypothetical protein |
|  | CF575522 | similar to zinc finger protein |
| novel_mir_32 | CF570081 | similar to nucleolin |
|  | CF576411 | similar to cytochrome P450 |

The Sugarcane_Unigene database (65,852 unigenes) established by our previous transcriptome analysis in ROC22 and YA05-179 post-*S. scitamineum* infection for 24 h, 48 h and 120 h [47] and the sugarcane EST in GenBank were used as search databases to predict the target genes of the known miRNAs and the novel miRNAs.
