# Supplementary material for: Small RNA sequencing reveals a role for sugarcane miRNAs and their targets in response to Sporisorium scitamineum infection
Source: BMC Genomics. 2017 Apr 24;18:325. doi: 10.1186/s12864-017-3716-4 (PMC5404671; doi:10.1186/s12864-017-3716-4)
Supplement: Supplementary file 17 — KEGG analysis of predicted target genes of known miRNAs in RT/RCK. (DOC 58 kb) [file 12864_2017_3716_MOESM17_ESM.doc]

**Table S13. KEGG analysis of predicted target genes of known miRNAs in RT/RCK**

| **NO.** | **Pathway** | **Target genes with pathway annotation (353)** | **All genes of the species with pathway annotation (161369)** | **P value** | **Q value** | **Pathway ID** |
| --- | --- | --- | --- | --- | --- | --- |
| 1 | [Pathogenic](../../../../../%E5%91%B5%E6%A3%89/2%20PB/13%20%E7%8E%89%E5%8F%B6%E5%B0%8FRNA/%E5%8D%8E%E5%A4%A7%E6%95%B0%E6%8D%AE/%E7%94%98%E8%94%97%E9%BB%91%E7%A9%97%E7%97%85-%E5%B0%8FRNA%E6%B5%8B%E5%BA%8F/BGI_SmallRNA_report/Files/BGI_Function/known_miRNA_analysis/KO/R-48_R-0/R-48_R-0.htm#gene2) *Escherichia coli* infection | 73 (20.68%) | 5276 (3.27%) | 1.869E-36 | 1.673E-34 | ko05130 |
| 2 | [Phagosome](../../../../../%E5%91%B5%E6%A3%89/2%20PB/13%20%E7%8E%89%E5%8F%B6%E5%B0%8FRNA/%E5%8D%8E%E5%A4%A7%E6%95%B0%E6%8D%AE/%E7%94%98%E8%94%97%E9%BB%91%E7%A9%97%E7%97%85-%E5%B0%8FRNA%E6%B5%8B%E5%BA%8F/BGI_SmallRNA_report/Files/BGI_Function/known_miRNA_analysis/KO/R-48_R-0/R-48_R-0.htm#gene3) | 73 (20.68%) | 5569 (3.45%) | 5.909E-35 | 3.526E-33 | ko04145 |
| 3 | [Nucleotide excision repair](../../../../../%E5%91%B5%E6%A3%89/2%20PB/13%20%E7%8E%89%E5%8F%B6%E5%B0%8FRNA/%E5%8D%8E%E5%A4%A7%E6%95%B0%E6%8D%AE/%E7%94%98%E8%94%97%E9%BB%91%E7%A9%97%E7%97%85-%E5%B0%8FRNA%E6%B5%8B%E5%BA%8F/BGI_SmallRNA_report/Files/BGI_Function/known_miRNA_analysis/KO/R-48_R-0/R-48_R-0.htm#gene5) | 28 (7.93%) | 1513 (0.94%) | 1.681E-17 | 6.017E-16 | ko03420 |
| 4 | [DNA replication](../../../../../%E5%91%B5%E6%A3%89/2%20PB/13%20%E7%8E%89%E5%8F%B6%E5%B0%8FRNA/%E5%8D%8E%E5%A4%A7%E6%95%B0%E6%8D%AE/%E7%94%98%E8%94%97%E9%BB%91%E7%A9%97%E7%97%85-%E5%B0%8FRNA%E6%B5%8B%E5%BA%8F/BGI_SmallRNA_report/Files/BGI_Function/known_miRNA_analysis/KO/R-48_R-0/R-48_R-0.htm#gene6) | 28 (7.93%) | 2135 (1.32%) | 8.242E-14 | 2.459E-12 | ko03030 |
| 5 | [Homologous recombination](../../../../../%E5%91%B5%E6%A3%89/2%20PB/13%20%E7%8E%89%E5%8F%B6%E5%B0%8FRNA/%E5%8D%8E%E5%A4%A7%E6%95%B0%E6%8D%AE/%E7%94%98%E8%94%97%E9%BB%91%E7%A9%97%E7%97%85-%E5%B0%8FRNA%E6%B5%8B%E5%BA%8F/BGI_SmallRNA_report/Files/BGI_Function/known_miRNA_analysis/KO/R-48_R-0/R-48_R-0.htm#gene7) | 28 (7.93%) | 2223 (1.38%) | 2.164E-13 | 5.534E-12 | ko03440 |
| 6 | [Mismatch repair](../../../../../%E5%91%B5%E6%A3%89/2%20PB/13%20%E7%8E%89%E5%8F%B6%E5%B0%8FRNA/%E5%8D%8E%E5%A4%A7%E6%95%B0%E6%8D%AE/%E7%94%98%E8%94%97%E9%BB%91%E7%A9%97%E7%97%85-%E5%B0%8FRNA%E6%B5%8B%E5%BA%8F/BGI_SmallRNA_report/Files/BGI_Function/known_miRNA_analysis/KO/R-48_R-0/R-48_R-0.htm#gene8) | 28 (7.93%) | 3334 (2.07%) | 2.194E-09 | 4.910E-08 | ko03430 |
| 7 | [Stilbenoid, diarylheptanoid and gingerol biosynthesis](../../../../../%E5%91%B5%E6%A3%89/2%20PB/13%20%E7%8E%89%E5%8F%B6%E5%B0%8FRNA/%E5%8D%8E%E5%A4%A7%E6%95%B0%E6%8D%AE/%E7%94%98%E8%94%97%E9%BB%91%E7%A9%97%E7%97%85-%E5%B0%8FRNA%E6%B5%8B%E5%BA%8F/BGI_SmallRNA_report/Files/BGI_Function/known_miRNA_analysis/KO/R-48_R-0/R-48_R-0.htm#gene11) | 11 (3.12%) | 1393 (0.86%) | 0.0003 | 4.882E-03 | ko00945 |
| 8 | [MAPK signaling pathway](../../../../../%E5%91%B5%E6%A3%89/2%20PB/13%20%E7%8E%89%E5%8F%B6%E5%B0%8FRNA/%E5%8D%8E%E5%A4%A7%E6%95%B0%E6%8D%AE/%E7%94%98%E8%94%97%E9%BB%91%E7%A9%97%E7%97%85-%E5%B0%8FRNA%E6%B5%8B%E5%BA%8F/BGI_SmallRNA_report/Files/BGI_Function/known_miRNA_analysis/KO/R-48_R-0/R-48_R-0.htm#gene12) | 13 (3.68%) | 1896 (1.17%) | 0.0003 | 5.052E-03 | ko04010 |
| 9 | [Other types of O-glycan biosynthesis](../../../../../%E5%91%B5%E6%A3%89/2%20PB/13%20%E7%8E%89%E5%8F%B6%E5%B0%8FRNA/%E5%8D%8E%E5%A4%A7%E6%95%B0%E6%8D%AE/%E7%94%98%E8%94%97%E9%BB%91%E7%A9%97%E7%97%85-%E5%B0%8FRNA%E6%B5%8B%E5%BA%8F/BGI_SmallRNA_report/Files/BGI_Function/known_miRNA_analysis/KO/R-48_R-0/R-48_R-0.htm#gene14) | 3 (0.85%) | 108 (0.07%) | 0.0018 | 2.285E-02 | ko00514 |
| 10 | [Cutin, suberine and wax biosynthesis](../../../../../%E5%91%B5%E6%A3%89/2%20PB/13%20%E7%8E%89%E5%8F%B6%E5%B0%8FRNA/%E5%8D%8E%E5%A4%A7%E6%95%B0%E6%8D%AE/%E7%94%98%E8%94%97%E9%BB%91%E7%A9%97%E7%97%85-%E5%B0%8FRNA%E6%B5%8B%E5%BA%8F/BGI_SmallRNA_report/Files/BGI_Function/known_miRNA_analysis/KO/R-48_R-0/R-48_R-0.htm#gene15) | 6 (1.7%) | 619 (0.38%) | 0.0026 | 3.123E-02 | ko00073 |
| 11 | [Plant hormone signal transduction](../../../../../%E5%91%B5%E6%A3%89/2%20PB/13%20%E7%8E%89%E5%8F%B6%E5%B0%8FRNA/%E5%8D%8E%E5%A4%A7%E6%95%B0%E6%8D%AE/%E7%94%98%E8%94%97%E9%BB%91%E7%A9%97%E7%97%85-%E5%B0%8FRNA%E6%B5%8B%E5%BA%8F/BGI_SmallRNA_report/Files/BGI_Function/known_miRNA_analysis/KO/R-48_R-0/R-48_R-0.htm#gene16) | 25 (7.08%) | 7173 (4.45%) | 0.0159 | 1.776E-01 | ko04075 |
| 12 | [Base excision repair](../../../../../%E5%91%B5%E6%A3%89/2%20PB/13%20%E7%8E%89%E5%8F%B6%E5%B0%8FRNA/%E5%8D%8E%E5%A4%A7%E6%95%B0%E6%8D%AE/%E7%94%98%E8%94%97%E9%BB%91%E7%A9%97%E7%97%85-%E5%B0%8FRNA%E6%B5%8B%E5%BA%8F/BGI_SmallRNA_report/Files/BGI_Function/known_miRNA_analysis/KO/R-48_R-0/R-48_R-0.htm#gene17) | 8 (2.27%) | 1690 (1.05%) | 0.0341 | 3.399E-01 | ko03410 |
| 13 | [N-Glycan biosynthesis](../../../../../%E5%91%B5%E6%A3%89/2%20PB/13%20%E7%8E%89%E5%8F%B6%E5%B0%8FRNA/%E5%8D%8E%E5%A4%A7%E6%95%B0%E6%8D%AE/%E7%94%98%E8%94%97%E9%BB%91%E7%A9%97%E7%97%85-%E5%B0%8FRNA%E6%B5%8B%E5%BA%8F/BGI_SmallRNA_report/Files/BGI_Function/known_miRNA_analysis/KO/R-48_R-0/R-48_R-0.htm#gene20) | 4 (1.13%) | 600 (0.37%) | 0.0438 | 3.925E-01 | ko00510 |

RCK and RT: ROC22 under sterile water and *Sporisorium scitamineum* stress after 48 h, respectively.
