# Supplementary material for: Small RNA sequencing reveals a role for sugarcane miRNAs and their targets in response to Sporisorium scitamineum infection
Source: BMC Genomics. 2017 Apr 24;18:325. doi: 10.1186/s12864-017-3716-4 (PMC5404671; doi:10.1186/s12864-017-3716-4)
Supplement: Supplementary file 18 — KEGG analysis of predicted target genes of known miRNAs in YAT/YACK. (DOC 34 kb) [file 12864_2017_3716_MOESM18_ESM.doc]

**Table S14. KEGG analysis of predicted target genes of known miRNAs in YAT/YACK**

| **NO.** | **Pathway** | **Target genes with pathway annotation (353)** | **All genes of the species with pathway annotation (161369)** | **P value** | **Q value** | **Pathway ID** |
| --- | --- | --- | --- | --- | --- | --- |
| 1 | [Pathogenic](../../../../../%E5%91%B5%E6%A3%89/2%20PB/13%20%E7%8E%89%E5%8F%B6%E5%B0%8FRNA/%E5%8D%8E%E5%A4%A7%E6%95%B0%E6%8D%AE/%E7%94%98%E8%94%97%E9%BB%91%E7%A9%97%E7%97%85-%E5%B0%8FRNA%E6%B5%8B%E5%BA%8F/BGI_SmallRNA_report/Files/BGI_Function/known_miRNA_analysis/KO/YA-48_YA-0/YA-48_YA-0.htm#gene2) *Escherichia coli* infection | 25 (39.06%) | 5276 (3.27%) | 8.004E-21 | 2.721E-19 | ko05130 |
| 2 | [Phagosome](../../../../../%E5%91%B5%E6%A3%89/2%20PB/13%20%E7%8E%89%E5%8F%B6%E5%B0%8FRNA/%E5%8D%8E%E5%A4%A7%E6%95%B0%E6%8D%AE/%E7%94%98%E8%94%97%E9%BB%91%E7%A9%97%E7%97%85-%E5%B0%8FRNA%E6%B5%8B%E5%BA%8F/BGI_SmallRNA_report/Files/BGI_Function/known_miRNA_analysis/KO/YA-48_YA-0/YA-48_YA-0.htm#gene3) | 25 (39.06%) | 5569 (3.45%) | 2.890E-20 | 6.551E-19 | ko04145 |
| 3 | [Other types of O-glycan biosynthesis](../../../../../%E5%91%B5%E6%A3%89/2%20PB/13%20%E7%8E%89%E5%8F%B6%E5%B0%8FRNA/%E5%8D%8E%E5%A4%A7%E6%95%B0%E6%8D%AE/%E7%94%98%E8%94%97%E9%BB%91%E7%A9%97%E7%97%85-%E5%B0%8FRNA%E6%B5%8B%E5%BA%8F/BGI_SmallRNA_report/Files/BGI_Function/known_miRNA_analysis/KO/YA-48_YA-0/YA-48_YA-0.htm#gene4) | 3 (4.69%) | 108 (0.07%) | 1.179E-05 | 2.004E-04 | ko00514 |
| 4 | [Cell cycle](../../../../../%E5%91%B5%E6%A3%89/2%20PB/13%20%E7%8E%89%E5%8F%B6%E5%B0%8FRNA/%E5%8D%8E%E5%A4%A7%E6%95%B0%E6%8D%AE/%E7%94%98%E8%94%97%E9%BB%91%E7%A9%97%E7%97%85-%E5%B0%8FRNA%E6%B5%8B%E5%BA%8F/BGI_SmallRNA_report/Files/BGI_Function/known_miRNA_analysis/KO/YA-48_YA-0/YA-48_YA-0.htm#gene5) | 2 (3.13%) | 212 (0.13%) | 0.0033 | 4.463E-02 | ko04112 |

YACK and YAT: YA05-179 under sterile water and *Sporisorium scitamineum* stress after 48 h, respectively.
