# Supplementary material for: Small RNA sequencing reveals a role for sugarcane miRNAs and their targets in response to Sporisorium scitamineum infection
Source: BMC Genomics. 2017 Apr 24;18:325. doi: 10.1186/s12864-017-3716-4 (PMC5404671; doi:10.1186/s12864-017-3716-4)
Supplement: Supplementary file 19 — KEGG analysis of predicted target genes of novel miRNAs in RT/RCK. (DOC 57 kb) [file 12864_2017_3716_MOESM19_ESM.doc]

**Table S15. KEGG analysis of predicted target genes of novel miRNAs in RT/RCK**

| **NO.** | **Pathway** | **Target genes with pathway annotation (168)** | **All genes of the species with pathway annotation (161369)** | **P value** | **Q value** | **Pathway ID** |
| --- | --- | --- | --- | --- | --- | --- |
| 1 | [Apoptosis](../../../../../%E5%91%B5%E6%A3%89/2%20PB/13%20%E7%8E%89%E5%8F%B6%E5%B0%8FRNA/%E5%8D%8E%E5%A4%A7%E6%95%B0%E6%8D%AE/%E7%94%98%E8%94%97%E9%BB%91%E7%A9%97%E7%97%85-%E5%B0%8FRNA%E6%B5%8B%E5%BA%8F/BGI_SmallRNA_report/Files/BGI_Function/novel_miRNA_analysis/KO/R-48_R-0/R-48_R-0.htm#gene4) | 39 (23.21%) | 2067 (1.28%) | 5.545E-37 | 1.567E-35 | ko04210 |
| 2 | [Peroxisome](../../../../../%E5%91%B5%E6%A3%89/2%20PB/13%20%E7%8E%89%E5%8F%B6%E5%B0%8FRNA/%E5%8D%8E%E5%A4%A7%E6%95%B0%E6%8D%AE/%E7%94%98%E8%94%97%E9%BB%91%E7%A9%97%E7%97%85-%E5%B0%8FRNA%E6%B5%8B%E5%BA%8F/BGI_SmallRNA_report/Files/BGI_Function/novel_miRNA_analysis/KO/R-48_R-0/R-48_R-0.htm#gene6) | 35 (20.83%) | 1975 (1.22%) | 3.005E-32 | 5.660E-31 | ko04146 |
| 3 | [Cell cycle](../../../../../%E5%91%B5%E6%A3%89/2%20PB/13%20%E7%8E%89%E5%8F%B6%E5%B0%8FRNA/%E5%8D%8E%E5%A4%A7%E6%95%B0%E6%8D%AE/%E7%94%98%E8%94%97%E9%BB%91%E7%A9%97%E7%97%85-%E5%B0%8FRNA%E6%B5%8B%E5%BA%8F/BGI_SmallRNA_report/Files/BGI_Function/novel_miRNA_analysis/KO/R-48_R-0/R-48_R-0.htm#gene7) | 40 (23.81%) | 3187 (1.97%) | 3.587E-31 | 5.791E-30 | ko04110 |
| 4 | [RNA degradation](../../../../../%E5%91%B5%E6%A3%89/2%20PB/13%20%E7%8E%89%E5%8F%B6%E5%B0%8FRNA/%E5%8D%8E%E5%A4%A7%E6%95%B0%E6%8D%AE/%E7%94%98%E8%94%97%E9%BB%91%E7%A9%97%E7%97%85-%E5%B0%8FRNA%E6%B5%8B%E5%BA%8F/BGI_SmallRNA_report/Files/BGI_Function/novel_miRNA_analysis/KO/R-48_R-0/R-48_R-0.htm#gene8) | 21 (12.50%) | 3141 (1.95%) | 2.071E-11 | 2.925E-10 | ko03018 |
| 5 | [Non-homologous end-joining](../../../../../%E5%91%B5%E6%A3%89/2%20PB/13%20%E7%8E%89%E5%8F%B6%E5%B0%8FRNA/%E5%8D%8E%E5%A4%A7%E6%95%B0%E6%8D%AE/%E7%94%98%E8%94%97%E9%BB%91%E7%A9%97%E7%97%85-%E5%B0%8FRNA%E6%B5%8B%E5%BA%8F/BGI_SmallRNA_report/Files/BGI_Function/novel_miRNA_analysis/KO/R-48_R-0/R-48_R-0.htm#gene9) | 6 (3.57%) | 87 (0.05%) | 5.480E-10 | 6.543E-09 | ko03450 |
| 6 | [mRNA surveillance pathway](../../../../../%E5%91%B5%E6%A3%89/2%20PB/13%20%E7%8E%89%E5%8F%B6%E5%B0%8FRNA/%E5%8D%8E%E5%A4%A7%E6%95%B0%E6%8D%AE/%E7%94%98%E8%94%97%E9%BB%91%E7%A9%97%E7%97%85-%E5%B0%8FRNA%E6%B5%8B%E5%BA%8F/BGI_SmallRNA_report/Files/BGI_Function/novel_miRNA_analysis/KO/R-48_R-0/R-48_R-0.htm#gene10) | 20 (11.90%) | 3398 (2.11%) | 5.790E-10 | 6.543E-09 | ko03015 |
| 7 | [MAPK signaling pathway](../../../../../%E5%91%B5%E6%A3%89/2%20PB/13%20%E7%8E%89%E5%8F%B6%E5%B0%8FRNA/%E5%8D%8E%E5%A4%A7%E6%95%B0%E6%8D%AE/%E7%94%98%E8%94%97%E9%BB%91%E7%A9%97%E7%97%85-%E5%B0%8FRNA%E6%B5%8B%E5%BA%8F/BGI_SmallRNA_report/Files/BGI_Function/novel_miRNA_analysis/KO/R-48_R-0/R-48_R-0.htm#gene12) | 11 (6.55%) | 1099 (0.68%) | 2.844E-08 | 2.678E-07 | ko04011 |
| 8 | [RNA transport](../../../../../%E5%91%B5%E6%A3%89/2%20PB/13%20%E7%8E%89%E5%8F%B6%E5%B0%8FRNA/%E5%8D%8E%E5%A4%A7%E6%95%B0%E6%8D%AE/%E7%94%98%E8%94%97%E9%BB%91%E7%A9%97%E7%97%85-%E5%B0%8FRNA%E6%B5%8B%E5%BA%8F/BGI_SmallRNA_report/Files/BGI_Function/novel_miRNA_analysis/KO/R-48_R-0/R-48_R-0.htm#gene13) | 21 (12.50%) | 5840 (3.62%) | 8.889E-07 | 7.727E-06 | ko03013 |
| 9 | [Pantothenate and CoA biosynthesis](../../../../../%E5%91%B5%E6%A3%89/2%20PB/13%20%E7%8E%89%E5%8F%B6%E5%B0%8FRNA/%E5%8D%8E%E5%A4%A7%E6%95%B0%E6%8D%AE/%E7%94%98%E8%94%97%E9%BB%91%E7%A9%97%E7%97%85-%E5%B0%8FRNA%E6%B5%8B%E5%BA%8F/BGI_SmallRNA_report/Files/BGI_Function/novel_miRNA_analysis/KO/R-48_R-0/R-48_R-0.htm#gene14) | 7 (4.17%) | 670 (0.42%) | 7.641E-06 | 6.168E-05 | ko00770 |
| 10 | [Plant-pathogen interaction](../../../../../%E5%91%B5%E6%A3%89/2%20PB/13%20%E7%8E%89%E5%8F%B6%E5%B0%8FRNA/%E5%8D%8E%E5%A4%A7%E6%95%B0%E6%8D%AE/%E7%94%98%E8%94%97%E9%BB%91%E7%A9%97%E7%97%85-%E5%B0%8FRNA%E6%B5%8B%E5%BA%8F/BGI_SmallRNA_report/Files/BGI_Function/novel_miRNA_analysis/KO/R-48_R-0/R-48_R-0.htm#gene15) | 21 (12.50%) | 7682 (4.76%) | 5.500E-05 | 4.143E-04 | ko04626 |
| 11 | [Aminoacyl-tRNA biosynthesis](../../../../../%E5%91%B5%E6%A3%89/2%20PB/13%20%E7%8E%89%E5%8F%B6%E5%B0%8FRNA/%E5%8D%8E%E5%A4%A7%E6%95%B0%E6%8D%AE/%E7%94%98%E8%94%97%E9%BB%91%E7%A9%97%E7%97%85-%E5%B0%8FRNA%E6%B5%8B%E5%BA%8F/BGI_SmallRNA_report/Files/BGI_Function/novel_miRNA_analysis/KO/R-48_R-0/R-48_R-0.htm#gene16) | 6 (3.57%) | 1514 (0.94%) | 0.0053 | 3.768E-02 | ko00970 |
| 12 | [Brassinosteroid biosynthesis](../../../../../%E5%91%B5%E6%A3%89/2%20PB/13%20%E7%8E%89%E5%8F%B6%E5%B0%8FRNA/%E5%8D%8E%E5%A4%A7%E6%95%B0%E6%8D%AE/%E7%94%98%E8%94%97%E9%BB%91%E7%A9%97%E7%97%85-%E5%B0%8FRNA%E6%B5%8B%E5%BA%8F/BGI_SmallRNA_report/Files/BGI_Function/novel_miRNA_analysis/KO/R-48_R-0/R-48_R-0.htm#gene18) | 2 (1.19%) | 197 (0.12%) | 0.0182 | 1.144E-01 | ko00905 |
| 13 | [Homologous recombination](../../../../../%E5%91%B5%E6%A3%89/2%20PB/13%20%E7%8E%89%E5%8F%B6%E5%B0%8FRNA/%E5%8D%8E%E5%A4%A7%E6%95%B0%E6%8D%AE/%E7%94%98%E8%94%97%E9%BB%91%E7%A9%97%E7%97%85-%E5%B0%8FRNA%E6%B5%8B%E5%BA%8F/BGI_SmallRNA_report/Files/BGI_Function/novel_miRNA_analysis/KO/R-48_R-0/R-48_R-0.htm#gene19) | 6 (3.57%) | 2223 (1.38%) | 0.0297 | 1.764E-01 | ko03440 |

RCK and RT: ROC22 under sterile water and *Sporisorium scitamineum* stress after 48 h, respectively.
