# Supplementary material for: Small RNA sequencing reveals a role for sugarcane miRNAs and their targets in response to Sporisorium scitamineum infection
Source: BMC Genomics. 2017 Apr 24;18:325. doi: 10.1186/s12864-017-3716-4 (PMC5404671; doi:10.1186/s12864-017-3716-4)
Supplement: Supplementary file 20 — KEGG analysis of predicted target genes of novel miRNAs in YAT/YACK. (DOC 58 kb) [file 12864_2017_3716_MOESM20_ESM.doc]

**Table S16. KEGG analysis of predicted target genes of novel miRNAs in YAT/YACK**

| **NO.** | **Pathway** | **Target genes with pathway annotation (538)** | **All genes of the species with pathway annotation (161369)** | **P value** | **Q value** | **Pathway ID** |
| --- | --- | --- | --- | --- | --- | --- |
| 1 | [Plant-pathogen interaction](../../../../../%E5%91%B5%E6%A3%89/2%20PB/13%20%E7%8E%89%E5%8F%B6%E5%B0%8FRNA/%E5%8D%8E%E5%A4%A7%E6%95%B0%E6%8D%AE/%E7%94%98%E8%94%97%E9%BB%91%E7%A9%97%E7%97%85-%E5%B0%8FRNA%E6%B5%8B%E5%BA%8F/BGI_SmallRNA_report/Files/BGI_Function/novel_miRNA_analysis/KO/YA-48_YA-0/YA-48_YA-0.htm#gene1) | 71 (13.2%) | 7682 (4.76%) | 1.531E-14 | 3.444E-12 | ko04626 |
| 2 | [Stilbenoid, diarylheptanoid and gingerol biosynthesis](../../../../../%E5%91%B5%E6%A3%89/2%20PB/13%20%E7%8E%89%E5%8F%B6%E5%B0%8FRNA/%E5%8D%8E%E5%A4%A7%E6%95%B0%E6%8D%AE/%E7%94%98%E8%94%97%E9%BB%91%E7%A9%97%E7%97%85-%E5%B0%8FRNA%E6%B5%8B%E5%BA%8F/BGI_SmallRNA_report/Files/BGI_Function/novel_miRNA_analysis/KO/YA-48_YA-0/YA-48_YA-0.htm#gene11) | 14 (2.60%) | 1393 (0.86%) | 0.0003 | 6.309E-03 | ko00945 |
| 3 | [Calcium signaling pathway](../../../../../%E5%91%B5%E6%A3%89/2%20PB/13%20%E7%8E%89%E5%8F%B6%E5%B0%8FRNA/%E5%8D%8E%E5%A4%A7%E6%95%B0%E6%8D%AE/%E7%94%98%E8%94%97%E9%BB%91%E7%A9%97%E7%97%85-%E5%B0%8FRNA%E6%B5%8B%E5%BA%8F/BGI_SmallRNA_report/Files/BGI_Function/novel_miRNA_analysis/KO/YA-48_YA-0/YA-48_YA-0.htm#gene17) | 12 (2.23%) | 1319 (0.82%) | 0.0019 | 2.465E-02 | ko04020 |
| 4 | [Polycyclic aromatic hydrocarbon degradation](../../../../../%E5%91%B5%E6%A3%89/2%20PB/13%20%E7%8E%89%E5%8F%B6%E5%B0%8FRNA/%E5%8D%8E%E5%A4%A7%E6%95%B0%E6%8D%AE/%E7%94%98%E8%94%97%E9%BB%91%E7%A9%97%E7%97%85-%E5%B0%8FRNA%E6%B5%8B%E5%BA%8F/BGI_SmallRNA_report/Files/BGI_Function/novel_miRNA_analysis/KO/YA-48_YA-0/YA-48_YA-0.htm#gene23) | 7 (1.30%) | 692 (0.43%) | 0.0092 | 8.671E-02 | ko00624 |
| 5 | [Phenylalanine metabolism](../../../../../%E5%91%B5%E6%A3%89/2%20PB/13%20%E7%8E%89%E5%8F%B6%E5%B0%8FRNA/%E5%8D%8E%E5%A4%A7%E6%95%B0%E6%8D%AE/%E7%94%98%E8%94%97%E9%BB%91%E7%A9%97%E7%97%85-%E5%B0%8FRNA%E6%B5%8B%E5%BA%8F/BGI_SmallRNA_report/Files/BGI_Function/novel_miRNA_analysis/KO/YA-48_YA-0/YA-48_YA-0.htm#gene24) | 16 (2.97%) | 2451 (1.52%) | 0.0092 | 8.671E-02 | ko00360 |
| 6 | [Bisphenol degradation](../../../../../%E5%91%B5%E6%A3%89/2%20PB/13%20%E7%8E%89%E5%8F%B6%E5%B0%8FRNA/%E5%8D%8E%E5%A4%A7%E6%95%B0%E6%8D%AE/%E7%94%98%E8%94%97%E9%BB%91%E7%A9%97%E7%97%85-%E5%B0%8FRNA%E6%B5%8B%E5%BA%8F/BGI_SmallRNA_report/Files/BGI_Function/novel_miRNA_analysis/KO/YA-48_YA-0/YA-48_YA-0.htm#gene25) | 7 (1.30%) | 706 (0.44%) | 0.0102 | 9.186E-02 | ko00363 |
| 7 | [Apoptosis](../../../../../%E5%91%B5%E6%A3%89/2%20PB/13%20%E7%8E%89%E5%8F%B6%E5%B0%8FRNA/%E5%8D%8E%E5%A4%A7%E6%95%B0%E6%8D%AE/%E7%94%98%E8%94%97%E9%BB%91%E7%A9%97%E7%97%85-%E5%B0%8FRNA%E6%B5%8B%E5%BA%8F/BGI_SmallRNA_report/Files/BGI_Function/novel_miRNA_analysis/KO/YA-48_YA-0/YA-48_YA-0.htm#gene31) | 13 (2.42%) | 2067 (1.28%) | 0.0232 | 1.663E-01 | ko04210 |
| 8 | [Zeatin biosynthesis](../../../../../%E5%91%B5%E6%A3%89/2%20PB/13%20%E7%8E%89%E5%8F%B6%E5%B0%8FRNA/%E5%8D%8E%E5%A4%A7%E6%95%B0%E6%8D%AE/%E7%94%98%E8%94%97%E9%BB%91%E7%A9%97%E7%97%85-%E5%B0%8FRNA%E6%B5%8B%E5%BA%8F/BGI_SmallRNA_report/Files/BGI_Function/novel_miRNA_analysis/KO/YA-48_YA-0/YA-48_YA-0.htm#gene32) | 5 (0.93%) | 482 (0.30%) | 0.0236 | 1.663E-01 | ko00908 |
| 9 | [Meiosis](../../../../../%E5%91%B5%E6%A3%89/2%20PB/13%20%E7%8E%89%E5%8F%B6%E5%B0%8FRNA/%E5%8D%8E%E5%A4%A7%E6%95%B0%E6%8D%AE/%E7%94%98%E8%94%97%E9%BB%91%E7%A9%97%E7%97%85-%E5%B0%8FRNA%E6%B5%8B%E5%BA%8F/BGI_SmallRNA_report/Files/BGI_Function/novel_miRNA_analysis/KO/YA-48_YA-0/YA-48_YA-0.htm#gene33) | 9 (1.67%) | 1266 (0.78%) | 0.0279 | 1.867E-01 | ko04113 |
| 10 | [RNA polymerase](../../../../../%E5%91%B5%E6%A3%89/2%20PB/13%20%E7%8E%89%E5%8F%B6%E5%B0%8FRNA/%E5%8D%8E%E5%A4%A7%E6%95%B0%E6%8D%AE/%E7%94%98%E8%94%97%E9%BB%91%E7%A9%97%E7%97%85-%E5%B0%8FRNA%E6%B5%8B%E5%BA%8F/BGI_SmallRNA_report/Files/BGI_Function/novel_miRNA_analysis/KO/YA-48_YA-0/YA-48_YA-0.htm#gene35) | 9 (1.67%) | 1277 (0.79%) | 0.0293 | 1.867E-01 | ko03020 |
| 11 | [Plant hormone signal transduction](../../../../../%E5%91%B5%E6%A3%89/2%20PB/13%20%E7%8E%89%E5%8F%B6%E5%B0%8FRNA/%E5%8D%8E%E5%A4%A7%E6%95%B0%E6%8D%AE/%E7%94%98%E8%94%97%E9%BB%91%E7%A9%97%E7%97%85-%E5%B0%8FRNA%E6%B5%8B%E5%BA%8F/BGI_SmallRNA_report/Files/BGI_Function/novel_miRNA_analysis/KO/YA-48_YA-0/YA-48_YA-0.htm#gene39) | 33 (6.13%) | 7173 (4.45%) | 0.0410 | 2.364E-01 | ko04075 |
| 12 | [Glucosinolate biosynthesis](../../../../../%E5%91%B5%E6%A3%89/2%20PB/13%20%E7%8E%89%E5%8F%B6%E5%B0%8FRNA/%E5%8D%8E%E5%A4%A7%E6%95%B0%E6%8D%AE/%E7%94%98%E8%94%97%E9%BB%91%E7%A9%97%E7%97%85-%E5%B0%8FRNA%E6%B5%8B%E5%BA%8F/BGI_SmallRNA_report/Files/BGI_Function/novel_miRNA_analysis/KO/YA-48_YA-0/YA-48_YA-0.htm#gene40) | 3 (0.56%) | 243 (0.15%) | 0.0485 | 2.674E-01 | ko00966 |
| 13 | [Phenylpropanoid biosynthesis](../../../../../%E5%91%B5%E6%A3%89/2%20PB/13%20%E7%8E%89%E5%8F%B6%E5%B0%8FRNA/%E5%8D%8E%E5%A4%A7%E6%95%B0%E6%8D%AE/%E7%94%98%E8%94%97%E9%BB%91%E7%A9%97%E7%97%85-%E5%B0%8FRNA%E6%B5%8B%E5%BA%8F/BGI_SmallRNA_report/Files/BGI_Function/novel_miRNA_analysis/KO/YA-48_YA-0/YA-48_YA-0.htm#gene41) | 19 (3.53%) | 3742 (2.32%) | 0.0487 | 2.674E-01 | ko00940 |

YACK and YAT: YA05-179 under sterile water and *Sporisorium scitamineum* stress after 48 h, respectively.
